# Assessing the benefits of horizontal gene transfer by laboratory evolution and genome sequencing

**DOI:** 10.1186/s12862-018-1164-7

**Published:** 2018-04-19

**Authors:** Hoi Yee Chu, Kathleen Sprouffske, Andreas Wagner

**Affiliations:** 1 Institute of Evolutionary Biology and Environmental Studies, University of Zurich, Zurich, Switzerland; 2 The Swiss Institute of Bioinformatics, Quartier Sorge – Batiment Genopode, 1015 Lausanne, Switzerland; 3 Santa Fe Institute, Santa Fe, New Mexico USA

**Keywords:** Horizontal gene transfer, Experimental evolution, Novel nutrient adaptation

## Abstract

**Background:**

Recombination is widespread across the tree of life, because it helps purge deleterious mutations and creates novel adaptive traits. In prokaryotes, it often takes the form of horizontal gene transfer from a donor to a recipient bacterium. While such transfer is widespread in natural communities, its immediate fitness benefits are usually unknown. We asked whether any such benefits depend on the environment, and on the identity of donor and recipient strains. To this end, we adapted *Escherichia coli* to two novel carbon sources over several hundred generations of laboratory evolution, exposing evolving populations to various DNA donors.

**Results:**

At the end of these experiments, we measured fitness and sequenced the genomes of 65 clones from 34 replicate populations to study the genetic changes associated with adaptive evolution. Furthermore, we identified candidate de novo beneficial mutations. During adaptive evolution on the first carbon source, 4-Hydroxyphenylacetic acid (HPA), recombining populations adapted better, which was likely mediated by acquiring the *hpa* operon from the donor. In contrast, recombining populations did not adapt better to the second carbon source, butyric acid, even though they suffered fewer extinctions than non-recombining populations. The amount of DNA transferred, but not its benefit, strongly depended on the donor-recipient strain combination.

**Conclusions:**

To our knowledge, our study is the first to investigate the genomic consequences of prokaryotic recombination and horizontal gene transfer during laboratory evolution. It shows that the benefits of recombination strongly depend on the environment and the foreign DNA donor.

**Electronic supplementary material:**

The online version of this article (10.1186/s12862-018-1164-7) contains supplementary material, which is available to authorized users.

## Background

The recombination of genetic material that leads to the creation of novel and beneficial traits is achieved by different means in different organisms. In prokaryotes and their communities, it is achieved through bacterial conjugation, viral transduction, and transformation. All of these processes can lead to horizontal gene transfer, a prominent mode of recombination between bacterial genomes [1–3]. Such transfer occurs both between closely related and highly divergent species [4], and it can confer novel traits that help microbes adapt to a broad range of environments [5–7]. Horizontal gene transfer can involve DNA molecules that are circular or linear, single-stranded or double stranded, self-replicating or not [3, 8–10]. These molecules can be integrated into the host genome via homology-based or illegitimate recombination [11], whose by-products may include gene duplications [12] and large-scale structural genomic changes [13, 14]. Homologous recombination is the most common means for genomic integration of horizontally acquired genes [15]. It generally requires the RecBCD enzyme [16], which is highly conserved among bacteria [17]. Although homologous recombination may have originated as a DNA repair mechanism [18], it plays an important role in adaptive evolution, for example by inserting or replacing gene clusters that facilitate local adaptation [19]. In *E. coli,* recombination is no less frequent than spontaneous mutation [20–22], suggesting that recombination contributes substantially to genome evolution.

Horizontal gene transfer helps augment the genetic diversity of a microbial population or community by shuffling genes in the “flexible genome” [23], a part of the genome whose genes are often private to a locally adapted strain. Recent comparative studies of 2000 *E. coli* genomes indicate that the flexible genome may comprise thousands of different gene families, which may help *E. coli* to occupy a wide range of ecological niches [24].

Horizontally transferred genes typically have functions different from core housekeeping genes. They are lost and gained more readily than core genes [25–27], and can endow their recipient with new traits that facilitate adaptation to a changing environment [28, 29]. For example, the horizontal transfer of such genes has helped marine microbes adapt to a variety of carbon sources [19, 30], it has helped bacteria adapt to extreme environments [31, 32], and it has helped gut microbial communities or pathogens adapt to human hosts [33, 34]. A recent comparative studies of 53 *E. coli* genomes showed that at least 10% of adaptations to new environments may have been achieved by horizontal gene transfer [35].

Most evidence of recent horizontal gene transfer in prokaryotic communities comes from comparative genomics studies or phylogenetic reconstructions [36–38]. These often focus on horizontal transfer among phenotypically differentiated organisms of the same species, such as pathogenic and non-pathogenic strains [36, 39]. Such studies can help identify key horizontally transferred genes that confer novel traits, but they are inconclusive about the immediate fitness benefit (if any) of a horizontal gene transfer event, which may transfer one or few driver genes along with multiple passenger genes that may impose fitness costs on host [40]. Such fitness information can be provided by laboratory evolution experiments [41, 42]. However, there are few such experiments that study horizontal gene transfer [43–49], and even fewer that do not focus on the transfer of plasmids [46–48] but of chromosomal genes [41–43]. In one of the latter experiments [43], *E. coli* K12 adapted to a constant glucose minimum environment while recombining with *E. coli* B REL606. Although recombination conferred increased genetic diversity, it did not improve growth significantly. In a more recent experiment, replacing ribosomal protein coding genes of *Salmonella typhimurium* with orthologues from other eubacteria, yeast, or archaea resulted in poor fitnesses due to suboptimal expression of these foreign genes [44]. However, after 40-250 genereations of laboratory evolution, fitness improved through amplification of the affected genes. In a third experiment [45], *Salmonella* transformed with random chromosomal DNA fragments from *Bacteroides fragilis*, *Proteus mirabilis*, and human intestinal phages showed reduced fitness in a constant glucose minmum environment, suggesting that horizontal gene transfer can be costly. Neither of these experiments demonstrated a direct advantage of horizontal gene transfer observed in natural populations [5, 50].

We here conducted laboratory evolution experiments that aimed to address several fundamental questions. Can the advantages of horizontal gene transfer be demonstrated on the short time scales of laboratory evolution? And if so, what is the genetic basis of specific adaptive changes brought about by horizontal gene transfer in evolution experiments? To address these and related questions, we conducted two evolution experiments that expose DNA recipient strains of *E.coli* to various donor strains that can transfer DNA to them, and that select for the recipient’s viability on a novel carbon and energy source. In the first experiment, we used a carbon source on which the donor could grow, but the recipient could not, such that horizontal transfer and recombination would be required for growth of the recipient. In the second experiment we used a carbon source on which neither donor nor recipient could grow, such that a combination of recombination and point mutations might be needed to ensure the recipient’s viability.

At the end of the evolution experiments, we measured the growth phenotypes of evolving populations, and analysed the complete genomes of 65 clones from these populations. Our observations show that the advantage of horizontal gene transfer depends critically on the growth environment, and less so on the donor strain. Horizontal gene transfer was the key driver for adaptation on HPA, whereas a combination of point mutations and horizontal transfer events may have facilitated adaptation on butyric acid.

## Results

### Experimental design

Our donors and recipients are derived from *E. coli* K12, B, and W strains (Additional file 1: Table S1), which originated from different *E. coli* subspecies. *E. coli* K12 and B are most closely related (0.8% nucleotide divergence among orthologous genes, 19.42% of genes non-shared between the strains. Additional file 2: Table S2). *E. coli W* differs to a greater extent from both *E. coli* K12 and B [51] (1.3 and 1.4% nucleotide divergence, respectively, 26% of non-shared genes, Additional file 2: Table S2).

To identify suitable carbon sources for experimental evolution, we used available experimental data from BIOLOG phenotyping microarrays [51–53] and computational modeling using flux balance analysis [54] of our strains’ metabolisms (Methods). Two such carbon sources emerged from this analysis. The first of them is 4-Hydroxyphenylacetate (HPA). *E. coli* K12 is unable to grow on this carbon source, but the B and W strain are able to grow in it, probably because they harbor the *hpa* operon [55]. The *hpa* operon encodes 11 gene products that import and metabolize HPA and structurally related chemicals [55]. Two of these products, *hpaC* and *hpaB,* form a two component 4-hydroxyphenylacetate 3-hydroxylase and are responsible for the first step of HPA degradation. Although the K12 strain lacks the *hpa* operon, it harbors the *paa* operon for the degradation of phenylacetate (PA). HPA is a hydroxylated derivative of PA [56, 57]. We reasoned that recombinational integration of the *hpa* operon into the recipient strain or point mutations that enable the recipient to convert HPA to PA, may be sufficient to convey the ability to grow on HPA.

**Fig. 1:**
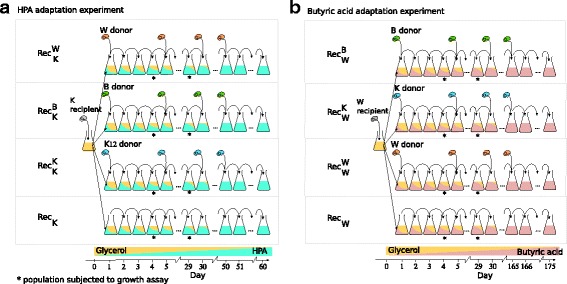
Experimental design of both evolution experiments. **a** We evolved multiple replicate populations of the K recipient strain on HPA for 60 serial transfer cycles (< 400 generations). Specifically, we established populations that we periodically exposed to W donor cells (6 replicate populations, $\operatorname{Re}{\mathrm{c}}_{\mathrm{K}}^{\mathrm{W}}$), to B donor cells (6 replicates, $\operatorname{Re}{\mathrm{c}}_{\mathrm{K}}^{\mathrm{B}}$), to K donor cells (6 replicates, $\operatorname{Re}{\mathrm{c}}_{\mathrm{K}}^{\mathrm{K}}$), and to no donor cells (6 replicates, Rec_K_ see Methods). **b** We evolved multiple replicate populations of the W recipient strain on butyric acid for 175 days (~ 1155 generations). Specifically, we established populations that we periodically exposed to B donor cells (6 replicate populations, $\operatorname{Re}{\mathrm{c}}_{\mathrm{W}}^{\mathrm{B}}$), to K donor cells (6 replicates, $\operatorname{Re}{\mathrm{c}}_{\mathrm{W}}^{\mathrm{K}}$), to W donor cells (6 replicates, $\operatorname{Re}{\mathrm{c}}_{\mathrm{W}}^{\mathrm{W}}$), and to no donor cells (6 replicates, Rec_W_). For both experiments, we seeded the recipient populations from a single overnight culture of the ancestral recipient (gray cell cartoon at the left of each panel) grown in glycerol. Everyday, we transferred each evolving population to fresh growth medium (Additional file 35: Table S4) by 100-fold dilution (black arrows). Every five days, we prepared glycerol stocks, screened for contaminations, monitored adaptation via growth assays, and added the appropriate donor to the evolving population (Methods). Over the course of the experiment, we gradually replaced glycerol (dark yellow) with HPA (cyan) (**a**) or butyric acid (pink) (**b**) in the growth medium, until only the novel carbon source was present. Then, we evolved the populations for 10 more days in the novel carbon source to ensure that the populations could grow exclusively on the novel carbon source

To find out, we conducted four different evolution experiments ($\operatorname{Re}{\mathrm{c}}_{\mathrm{K}}^{\mathrm{W}},\operatorname{Re}{\mathrm{c}}_{\mathrm{K}}^{\mathrm{B}},\operatorname{Re}{\mathrm{c}}_{\mathrm{K}}^{\mathrm{K}}$ and Rec_K_), each replicated six-fold. In the ${\mathrm{Rec}}_{\mathrm{K}}^{\mathrm{W}}$ experiment (Fig. 1), we exposed *E. coli* K12 recipients to a growth medium that shifted in the course of 400 generations (60 serial transfer cycles) from glycerol to HPA. During this time, we exposed the recipient every 33 generations (every five serial transfer cycles) to the *E.coli* W donor strain (Fig. 1a). The donor strain itself cannot grow in the experiment’s medium, because of a tryptophan auxotrophy (Additional file 1: Table S1, Additional file 3: Figure S1), and thus gradually gets diluted out of the culture over three transfer cycles (Additional file 4: Figure S4). During this time, however, it may transfer genes to the recipient that may help the recipient grow. During the experiment, we periodically checked for cross-contamination among replicates, verified that the donor had indeed not invaded the recipient culture (Additional file 5: Text S1), and determined the fraction of cells that were able to grow on HPA by plating (Methods). At the end of the experiment, we measured the growth rate of evolved populations, and of clones from these populations in HPA-containing liquid medium. We also sequenced the genomes of two clones per population to an average of 99.96-fold coverage.

To find out whether the donor’s identity matters for adaptive evolution, we performed the $\operatorname{Re}{\mathrm{c}}_{\mathrm{K}}^{\mathrm{B}}$ experiment in the same way as the $\operatorname{Re}{\mathrm{c}}_{\mathrm{K}}^{\mathrm{W}}$ experiment, with the exception that we exposed the recipient populations to the more closely related B donor strain. Finally, we also performed two control experiments, one in which the K recipient was exposed to an identical K donor strain (${\mathrm{Rec}}_{\mathrm{K}}^{\mathrm{K}}$), and one in which the recipient was not exposed to a donor (Rec_K_, Fig. 1a). At the end of each experiment, we again measured growth rates and sequenced the genomes of individual clones.

The second carbon source identified by our preliminary analyses was butyric acid (see Methods). Butyric acid is a short-chain fatty acid that can be utilized through the enzymes encoded by the *ato* and the *fad* operon [58]. Both operons are tightly regulated. They are released from catabolic repression only when preferred carbon sources (e.g. glucose) are exhausted [59]. The *fad* operon can be induced by long-chain fatty acids (more than 12 carbon atoms, C_12_) but not medium-chain (C_7-11_) or short-chain (C_2-4_) fatty acids [60]. Conversely, the *ato* operon is induced by short-chain fatty acids. Because both operons have to be activated to use short-chain fatty acids [61], *E. coli* can only degrade butyric acid during starvation, and in the presence of other long-chain fatty acids. Thus, *E. coli* generally cannot grow on butyric acid as its only carbon source. Additionally, butyric acid can be toxic to cells [54], and it lowers the pH of the growth medium, thus exposing the cells to acid stress [62, 63].

All our three strains encode the *fad* operon. In contrast, only the B and K12 strains harbor the *ato* operon. Although metabolic modeling predicts that the B and K12 strains are viable on butyric acid metabolism, because the necessary genes are present in the *fad* and *ato* operons, published BIOLOG [51, 53] and other growth data [54] show that none of our three ancestral strains can. This deficiency is likely caused by a combination of butyric acid toxicity and repression of the *fad* operon.

These observations motivate our choice of strain W as the recipient, and the other two strains as donors, reasoning that a combination of recombination (transfer of the *ato* operon to the W recipient) and point mutations may be needed to allow growth on butyric acid. More specifically, and analogous to the HPA experiment, we performed four different six-fold replicated experiments designated as ${\mathrm{Rec}}_{\mathrm{W}}^{\mathrm{K}}$, ${\mathrm{Rec}}_{\mathrm{W}}^{\mathrm{B}}$, ${\mathrm{Rec}}_{\mathrm{W}}^{\mathrm{W}}$, Rec_W_, followed by growth rate analysis and genome sequencing (Fig. 1b). These experiments lasted for 175 serial transfer cycles (~ 1155 generations). Of note, each donor strain in both the HPA and butyric acid experiments carries three origin of transfer (*OriT*) sequences (Additional file 1: Figure S1) to increase gene transfer efficiency [64].

### Evolutionary adaptation on HPA

After approximately 400 generations of laboratory evolution (Fig. 1a), 18 of our 4 × 6 = 24 replicate populations (Fig. 1a and Additional file 6: Figure S7) had adapted to survive on HPA. Three populations had become extinct and two further ${\mathrm{Rec}}_{\mathrm{K}}^{\mathrm{K}}$ and one Rec_K_ population showed evidence of contamination and were eliminated from further analyses (Additional file 5: Text S1 and Additional file 7: Figure S8).

**Fig. 2:**
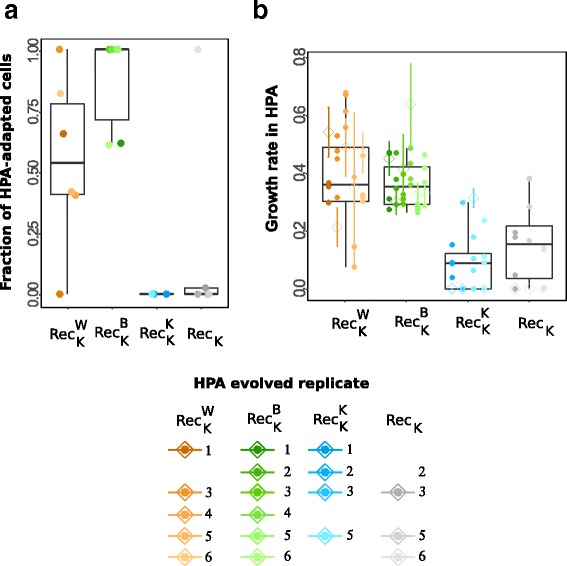
Adaptation of HPA evolved populations and clones. **a** Fraction of HPA-adapted cells (vertical axis) for each of our four (six-fold replicated) experimental treatments (horizontal axis), as determined by a plating assay (Methods). Solid circles indicate data from each individual population (color legend). Box whisker plots display the median (central bar), the first and the third quartile (top and bottom bar of the box), and the range (whiskers) of a 95% interval of the fraction of cells able to form colonies on HPA. **b** Mean population fitness of evolved populations (open diamonds, bars extend to one standard deviation from three biological replicates) and each of four clones isolated from each replicate population (solid circles, mean fitness from three biological replicates), measured as growth rate in liquid medium supplemented with HPA. Box whisker plots display the median of the mean fitness of clones (center bar), the first and third quartiles (box boundaries), and the range of a 95% interval of the data (whiskers). '$\operatorname{Re}{\mathrm{c}}_{\mathrm{Y}}^{\mathrm{X}}$' denotes a population of Y recipients exposed to donor X. Each replicate population within a treatment is labeled with a number and a distinct color in the legend. We note that the ancestors could not grow in HPA (Additional file 28: Figure S3), and fitness can thus not be given relative to the ancestor. Data is not shown for populations that had gone extinct during the experiment, and for clones that showed signs of contamination (Additional file 23: Text S3)

We characterized the ability of the remaining 18 populations to grow on HPA with three complementary assays. The first assay is based on the fraction of a population’s cells that can form colonies on plates which contain HPA as the sole carbon source (Methods). In this assay, our two control experiments (Rec_K_ and ${\mathrm{Rec}}_{\mathrm{K}}^{\mathrm{K}}$, one without a donor and the other with a donor identical to the recipient) showed a statistically indistinguishable fraction of HPA-adapted cells (Fig. 2a and Additional file 8: Table S7, test 1, Mann-Whitney U-test, *p* = 0.43). Thus, if the donor is identical to the recipient, recombination and horizontal gene transfer do not provide an advantage. However, this was not the case when the donor differed from the recipient (${\mathrm{Rec}}_{\mathrm{K}}^{\mathrm{W}}$ and ${\mathrm{Rec}}_{\mathrm{K}}^{\mathrm{B}}$). In both experiments, the evolving populations showed a significantly greater fraction of HPA-adapted cell than the ${\mathrm{Rec}}_{\mathrm{K}}^{\mathrm{K}}$ control (Fig. 2a and Additional file 8: Table S7, ${\mathrm{Rec}}_{\mathrm{K}}^{\mathrm{W}}$: test 2, Mann-Whitney Utest, *p* = 0.0079; ${\mathrm{Rec}}_{\mathrm{K}}^{\mathrm{B}}$: test 3, Mann-Whitney U-test, *p* = 0.0048). The fraction of HPA-adapted cells in the two (${\mathrm{Rec}}_{\mathrm{K}}^{\mathrm{W}}$ and ${\mathrm{Rec}}_{\mathrm{K}}^{\mathrm{B}}$) experiments was statistically indistinguishable from one another (Additional file 8: Table S7, test 4, Mann-Whitney U-test, *p* = 0.089).

In the second assay, we determined the growth of evolved populations in liquid culture on HPA as an indicator of fitness. More specifically, we determined the growth rate during a 48-h growth cycle. We found that recipients exposed to a donor ($\operatorname{Re}{\mathrm{c}}_{\mathrm{K}}^{\mathrm{W}}$, $\operatorname{Re}{\mathrm{c}}_{\mathrm{K}}^{\mathrm{B}}$ or $\operatorname{Re}{\mathrm{c}}_{\mathrm{K}}^{\mathrm{K}}$) grew better than recipients exposed to no donor (Rec_K_). In addition, recipients exposed to a donor different from themselves ($\operatorname{Re}{\mathrm{c}}_{\mathrm{K}}^{\mathrm{W}},\operatorname{Re}{\mathrm{c}}_{\mathrm{K}}^{\mathrm{B}}$) grew better than recipients exposed to the same donor (${\mathrm{Rec}}_{\mathrm{K}}^{\mathrm{K}};$ Fig. 2B, Additional file 8: Table S7, tests 5-6) and recipients without donors (Rec_K_; Fig. 2b and Additional file 8: Table S7, tests 7-8). In contrast, whether recipients were exposed to either B or W donors did not affect the final growth phenotype (Fig. 2b and Additional file 8: Table S7, test 9, Mann-Whitney U-test, *p* = 0.16).

Finally, we also repeated this growth analysis for four random clones isolated from each of our 18 populations. In these measurements, recipients exposed to an identical donor ($\operatorname{Re}{\mathrm{c}}_{\mathrm{K}}^{\mathrm{K}}$) grew poorly, at a rate similar to recipients exposed to no donor (Rec_K_) (Fig. 2b and Additional file 8: Table S7, test 10, Mann-Whitney U-test, *p* = 0.92). In contrast, recipients exposed to a different donor grew much faster, regardless of the donor (Fig. 2b and Additional file 8: Table S7, Additional file 9: Table S12). The fitness advantage conferred by the B donor was again similar to that conferred by the W donor (Additional file 8: Table S7, test 11 Mann-Whitney U-test, *p* = 0.41).

### Horizontal gene transfer drove HPA adaptation

We analysed genomes of 35 clones from 18 HPA-evolved replicate populations and the donor and recipient ancestors, with the purpose to identify the incidence of horizontal gene transfer and its potential contribution to adaptation (see Methods and Additional file 10: Figure S14 for analytic workflow summary). We sequenced these genomes to a minimum of 41-fold and an average of 99-fold coverage (Additional file 11: Figure S5).

**Fig. 3:**
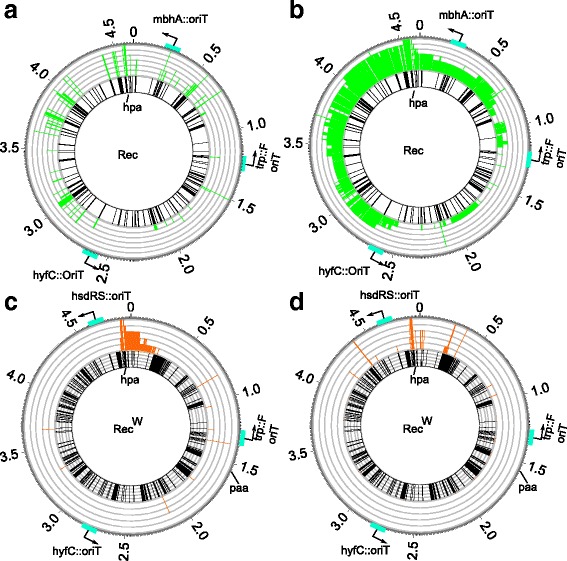
Circos plots of horizontally transferred genes in HPA-evolved clones. **a** Horizontally transferred genes among $\operatorname{Re}{\mathrm{c}}_{\mathrm{K}}^{\mathrm{B}}$ populations during HPA evolution. The circos plots show several concentric circles. The outermost circle (dark grey line) indicates genomic coordinates (in Mb) from the origin of replication (marked as 0), the location of the *oriT* located in the F-plasmid integrate, and the other two *oriT* sequences (blue rectangles). The innermost circle shows a radial black bar at each genomic location where a gene is present in the donor but not the recipient (K12) genome, as well as the location of the *hpa* operon in the *E*. *coli* REL606 B str. reference genome. The median circle (green bars) shows the number of populations that have acquired one or more genes from the B donor in at least one of the two clones sequenced (the maximum height of each green radial bar corresponds to six replicate populations). **b** Analogous to (**a**), except that the middle circle now reports the number of populations that have acquired genes (in *E.coli* B genome coordinates) that occur in both ancestral K recipient and the ancestral B donor. **c** and **d**, similar to (**a**) and (**b**), but for $\operatorname{Re}{\mathrm{c}}_{\mathrm{K}}^{\mathrm{W}}$ populations. The innermost circle shows a radial black bar at each genomic location where a gene is present in the *E. coli* W but not the K12 genome. The orange bars show the number of populations that have acquired genes from the W donor in at least one of the two sequenced clones, for genes (**c**) that occur only in the W donor, and (**d**) that occur in both donor and recipient. *Paa,* the operon responsible for degrading aromatic compounds [56]*,* is present in the W strain (see label at 4 o’clock) and the K strain, but not the B strain. All data are based on sequence coverage based estimation of horizontal gene transfer events (Methods)

In the six ${\mathrm{Rec}}_{\mathrm{K}}^{\mathrm{B}}$ populations, we observed that 2643 genes were transferred from the *E. coli* B donor to at least one K recipient clone (Fig. 3a and Additional file 12: Table S16). 91.18% (2410) of the transferred genes have an orthologue in the K recipient genome, a percentage that is not significantly greater than the 90.61% expected by chance alone, given that 368 genes do occur in the B genome but not in the K genome (out of 4937 genes surveyed using coverage-based and SNP polymorphism-based approaches, see Methods, Additional file 8: Table S7, test 12, Pearson χ^2^*p* = 0.67).

Overall, HPA evolved clones from recipient populations exposed to the B donor (Fig. 3a) contained 1.25-55.46% of horizontally transferred genes in their genomes (49-2159 out of 3893 surveyed genes suitable for identification of horizontal gene transfer using gene coverage or SNP polymorphisms, see Methods and Additional file 5: Text S1). These clones also had multiple recombination breakpoint regions (Additional file 13: Figure S9A), suggesting that multiple horizontal gene transfer events occurred. Breakpoints preferentially occurred in regions significantly enriched in repetitive DNA (Additional file 13: Figure S9E and Additional file 8: Table S7, test 13 and 14, Mann-Whitney U-test, *p* < 0.0026).

Motivated by the notion that homologous recombination often occurs in regions of high sequence similarity between the recipient and donor genomes [11], we further asked if breakpoint regions harbored fewer single nucleotide polymorphisms than randomly drawn genomic regions with the same length. However, no such significant differences exist (Additional file 8: Table S7, test 15, Mann-Whitney U-test, *p* = 0.45). A likely explanation is that the *E. coli* B and K12 genomes are very similar (Additional file 2: Table S2) across 92% of the genome [65]. Sequence divergence may thus pose few obstacles to recombination.

Horizontal gene transfer by bacterial conjugation typically starts at an *OriT* sequence, and transfers a contiguous stretch of DNA starting at this sequence [1]. In our experiments, the survival of transconjugants after transfer and genomic integration would be largely determined by natural selection and not genetic drift. The reason is that our populations were large, with bottleneck sizes of 2 × 10^6^ individuals as a result of periodic transfer (the population density before transfer was typically 1 × 10^8^/ml, yielding a bottleneck size of 20 μl × 1 × 10^8^ = 2 × 10^6^ individuals). The influence of selection is also evident from our sequence data: Contrary to the expectation that the incidence of observed transfer events declines with a gene’s distance from the *OriT,* we observe that the majority of retained transferred genes are far from any *OriT* sequence (mean distance: 841.35 kbp) (Additional file 13: Figure S9B)*.* However, we note that some repeatedly transferred genes (Fig. 3a) may attain high frequency by hitchhiking, because intragenomic recombination is rare in our genomes.

In contrast to our experiments with the B donor, fewer genes were transferred in our experiments with the W donor. Specifically, in the five $\operatorname{Re}{\mathrm{c}}_{\mathrm{K}}^{\mathrm{W}}$ populations, we observed that 319 genes were transferred from the *E. coli* W donor to at least one K recipient clone (Additional file 12: Table S16). 80.88% (258) of the transferred genes have an orthologue in the K recipient genome, a percentage that is not significantly greater than the 82.86% expected by chance alone, given that 811 genes do occur in the W genome but not in the K genome (Additional file 8: Table S7, test 16, Pearson χ^2^*p* = 0.37). Overall, evolved clones (Fig. 3b) harbored only between 0.90% and 7.12% horizontally transferred genes (35-287 of 5387 surveyed genes). Again, different clones, and clones from different replicate populations did not share the same recombination breakpoints (Additional file 13: Figure S9C). We observed that 96% (24 out of 25) of the breakpoints of the $\operatorname{Re}{\mathrm{c}}_{\mathrm{K}}^{\mathrm{W}}$ clones occurred in regions with no more than two repetitive elements, a density of repetitive DNA that is not different from that expected by chance alone (Additional file 8: Table S7, test 17, Mann-Whitney U-test, *p* = 0.17). We also observed that these breakpoints occurred in regions with SNP densities similar to randomly chosen regions from the *E. coli* K12 genome (Additional file 8: Table S7, test 18, Mann-Whitney U-test, *p* = 0.11).

Selection strongly influenced the retention of transferred genes, because, again, retained transferred genes were not preferentially closer to an *OriT* sequence, and generally occurred far away (871.21 kbp) from the nearest origin of transfer (Additional file 13: Figure S9D). The lower incidence of retained transferred genes for the W donor may be caused by the lower sequence similarity between the W donor and the K recipient, as compared to the B donor and the K recipient.

When we examined genes that were transferred from both the W and the B donor to the recipient, we found 222 such genes, 206 of which have orthologues in all three genomes. This number of transferred genes is significantly greater than expected by chance alone (Additional file 8: Table S7, test 19, randomization test, *p* = 7 × 10^− 5^).

All except one of the genes (*infA*) transferred from both the W and B donors (218) clustered in a 350 kbp region surrounding the origin of replication of both donor genomes (Fig. 3). In total, 17 genes were transferred to at least 90% of clones from both W and B donors (Additional file 14: Figure S10). Thirteen of these genes co-localised in a region that encompasses the 11 gene-*hpa* operon (Fig. 3 and Additional file 15: Figure S11), supporting the notion that transfer of the *hpa* operon is important for HPA utilization.

In contrast to the genes that were transferred from both the W and B donors, none of the genes transferred from only one (but not both) of these donors are likely to be associated with HPA utilization (Additional file 16: Text S2).

### No de novo mutations with obvious links to HPA metabolism

Next, we sought to identify de novo mutations that might also confer adaptation to HPA (Methods). To do so, we identified mutant (derived) alleles that occurred in the recipient genome and that originated in that genome. We discovered in total 35 such mutations (Additional file 17: Excel file S1), only three of which were synonymous. They fell within 21 recipient genes (Table 1, Additional file 18: Table S8), but none of these genes has a known function related to HPA or aromatic compound metabolism. Among them are *rpoB* and *rpo*C (Additional file 17: Excel file S1), which often experience beneficial mutations in laboratory evolution experiments [66–68]. De novo mutations in these and other genes may be involved in adaptation to the general experimental environment. We further examined de novo mutations in genes that had been transferred from the donor genomes. We found such mutations in three genes from the B donor and in six genes from the B donor, but none of these genes are known to be involved in HPA or aromatic compound metabolism. One $\operatorname{Re}{\mathrm{c}}_{\mathrm{K}}^{\mathrm{B}}$ population had a mutation in the horizontally acquired gene *spoT*, which is also frequently found as a target of positive selection in long-term evolution experiments [66–68].

**Table 1:** Number of de novo mutations located in protein-coding genes with functional annotations, for both the HPA-adapted and the butyric acid-adapted populations

Recipient	Donor	Total number of mutations discovered within protein coding genes with annotated functions	Number of mutant genes
Evolved on HPA			
K	W	78	30
K	B	16	6
K	K	21	7
K	–	26	11
Evolved on butyric acid			
W	B	7	6
W	K	2	2
W	W	22	19
W	–	25	12

### Evolutionary adaptation on butyric acid

In the second experiment, we evolved analogously 24 *E. coli* W recipient replicate populations for growth on butyric acid, with six replicates for each of four recombination conditions (Fig. 1b): In the first (${\mathrm{Rec}}_{\mathrm{W}}^{\mathrm{B}}$), we exposed populations of W recipients to B donors. In the second (${\mathrm{Rec}}_{\mathrm{W}}^{\mathrm{K}}$), we exposed populations of W recipients to K donors. We note that B and K donors show similar DNA divergence from the W recipient (Additional file 2: Table S2). In the third and fourth condition, we exposed populations of W recipients to W donors (${\mathrm{Rec}}_{\mathrm{W}}^{\mathrm{W}}$) or to no donors (Rec_W_).

### Recombination did not facilitate butyric acid adaptation

At the beginning of our experiment, no recipient cells were able to grow on butyric acid. At the end of the experiment (1155 generations, 175 days), 16 replicate populations had become able to do so (Additional file 19: Figure S12). One $\operatorname{Re}{\mathrm{c}}_{\mathrm{W}}^{\mathrm{B}}$ population, one $\operatorname{Re}{\mathrm{c}}_{\mathrm{W}}^{\mathrm{K}}$ population, three $\operatorname{Re}{\mathrm{c}}_{\mathrm{W}}^{\mathrm{W}}$ populations, and three Rec_W_ populations had gone extinct. The lower incidence of extinction in populations with different donors (17%) as opposed to populations with identical or no donors (50%) suggests an advantage of recombination for the survival of populations. More generally, butyric acid clearly poses substantial challenges for adaptation. This is evident not only from the substantial proportion of populations (8 of 24) that went extinct, but also from the observation that viability on butyric acid appeared very late in the experiment (beyond 800 generations; Additional file 19: Figure S12) for most surviving populations.

**Fig. 4:**
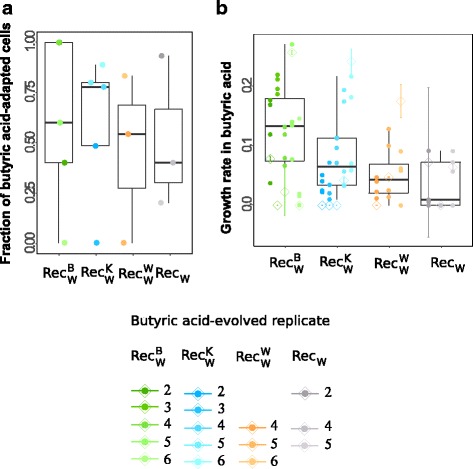
Adaptation to butyric acid. **a** Fraction of butyric acid-adapted cells (vertical axis) for each of our four (six-fold replicated) experimental treatments (horizontal axis), as determined by a plating assay (Methods). Solid circles indicate data from each individual population (color legend). Box whisker plots display the median (central bar), the first and the third quartile (top and bottom bar of the box), and the range (whiskers) of a 95% interval of the fraction of cells able to form colonies on HPA. **b** Mean fitness of evolved populations (open diamonds, bars extend to one standard deviation from three biological replicates), and each of four clones isolated from each replicate population (solid circles, mean fitness from three biological replicates), measured as the growth rate in liquid medium supplemented with butyric acid. Box whisker plots display the median of mean fitness of clones (center bar), the first and third quartiles (box boundaries), and the range of a 95% interval of the data (whiskers). '$\operatorname{Re}{\mathrm{c}}_{\mathrm{Y}}^{\mathrm{X}}$' denotes populations of Y recipients exposed to donor X. Each replicate population within a treatment is labeled with a number and a distinct color in the legend. We note that the ancestors could not grow in butyric acid (Additional file 28: Figure S3), and fitness can thus not be given relative to the ancestor. Data is not shown for populations that had gone extinct during the experiment

We again used three different assays to determine whether recombination provided a growth advantage for adaptation to butyric acid. It did not. First, in our plating assay (Fig. 4a), W recipient populations recombining with different donors reached a density of butyric acid-adapted clones that was statistically indistinguishable from populations with the same donor ($\operatorname{Re}{\mathrm{c}}_{\mathrm{W}}^{\mathrm{W}}$), and from populations without donor (Rec_W_; Additional file 8: Table S7, tests 20-25, Mann-Whitney U-test).

Second, when assessing the growth of evolved populations in liquid culture (Fig. 4b and Additional file 20: Table S13), we found that recipients exposed to different donors grow at rates that are very similar from those exposed to the same donor and to no donor (Additional file 8: Table S7, test 26-30). Finally, we also determined the growth of four clones isolated from each evolving population (Additional file 21: Table S14). Here, $\operatorname{Re}{\mathrm{c}}_{\mathrm{W}}^{\mathrm{B}}$ and $\operatorname{Re}{\mathrm{c}}_{\mathrm{W}}^{\mathrm{K}}$ clones showed statistically significantly higher fitness than Rec_W_ clones (Additional file 8: Table S7, test 31, Mann-Whitney U-test, *p* = 0.00068; test 32, Mann-Whitney U-test, *p* = 0.022; raw data in Additional file 21: Table S14). However, only $\operatorname{Re}{\mathrm{c}}_{\mathrm{W}}^{\mathrm{B}}$ clones but not $\operatorname{Re}{\mathrm{c}}_{\mathrm{W}}^{\mathrm{K}}$ clones grew significantly better than $\operatorname{Re}{\mathrm{c}}_{\mathrm{W}}^{\mathrm{W}}$ clones (Additional file 8: Table S7, test 33, Mann-Whitney U-test, *p* = 0.0018; test 34, Mann-Whitney U-test, *p* = 0.084). $\operatorname{Re}{\mathrm{c}}_{\mathrm{W}}^{\mathrm{B}}$ clones grew slightly but not significantly better than $\operatorname{Re}{\mathrm{c}}_{\mathrm{W}}^{\mathrm{K}}$ clones (Additional file 8: Table S7, test 35, Mann-Whitney U-test, *p* = 0.063).

### Horizontally transferred genes include the *ato* operon

To identify genetic changes associated with adaptation to butyric acid, we analysed whole-genome sequences of 30 clones from 15 evolved populations (minimum coverage 24-fold, average coverage 99-fold, Additional file 11: Figure S5) and of the ancestral donors and recipient (see Methods and Additional file 10: Figure S14 for analytic workflow summary).

Overall, the proportion of horizontally transferred genes was much smaller than in the HPA-adaptation experiment. In the $\operatorname{Re}{\mathrm{c}}_{\mathrm{W}}^{\mathrm{B}}$ populations, individual sequenced clones had acquired no more than 0.34% of all genes (8-13 genes out of 3821 surveyed genes suitable for horizontal gene transfer identification, see Methods) from the B donor. In total, only 22 genes were transferred from the B donor to at least one sequenced clone. Half of these genes (11 out of 22) encoded hypothetical proteins, and were scattered across the genome, with a pairwise distance of at least 40 kbp. Because the transferred regions were short, and often comprised only single genes, our approach was unable to identify recombination breakpoint regions. The small number of horizontally transferred genes may be due to the low conjugation efficiency of the B donor and the W recipient (3.84 × 10^− 10^, Additional file 5: Text S1).

**Fig. 5:**
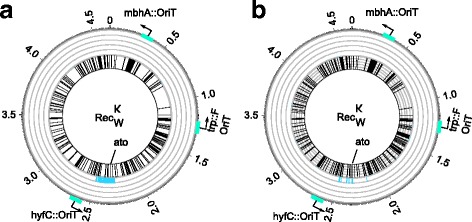
Circos plots of the distributions of horizontally transferred genes among butyric acid-evolved clones. **a** Putatively horizontally transferred genes among W recipients exposed to K donors in $\operatorname{Re}{\mathrm{c}}_{\mathrm{W}}^{\mathrm{K}}$ experiments during adaptive evolution on butyric acid. The circos plots show several concentric circles. The outermost circle (dark grey line) indicates genomic coordinates (in Mb) from the origin of replication (marked as 0), the location of the *oriT* located in the F-plasmid integrate, and the other two *oriT* sequences (green rectangles). The innermost circle shows a radial black bar at each genomic location where a gene is present in the ancestral donor (K) but not the ancestral recipient (W) genome (in K genome coordinates). The middle circle shows the number of populations that have acquired one or more genes in at least one of the sequenced clones of the population at that location in the K donor, as the height of each green radial bar (maximum height corresponding to five populations). **b** Analogous to (**a**), except that the middle circle now reports the number of sequenced clones that have acquired the gene at that location only for genes that occur both in the ancestral W recipient and the ancestral K donor. Note that all gene locations are in coordinates of the *E. coli* K12 reference genome. All data are based on sequence coverage based estimation of horizontally transferred genes (Methods). The location of the *ato* operon (at 2.32 Mb), which is involved in butyric acid degradation is marked at the innermost circle

In the $\operatorname{Re}{\mathrm{c}}_{\mathrm{W}}^{\mathrm{K}}$ populations, we only identified horizontally transferred genes in clones from one population (184 out of 5387 surveyed genes, Fig. 5, Additional file 12: Table S16). The transferred genes were on average far (325.27 kbp) away from the nearest *OriT* (Additional file 22: Figure S13B), indicating a role for selection in retaining them in the recipient genome*.* This population had experienced a large-scale horizontal gene transfer event (between 2.44 and 2.67 Mb of the W recipient genome, within gene *yejA* and near *yfdE*, Additional file 22: Figure S13A). The recombination breakpoints were unremarkable in terms of their repetitive DNA density (Additional file 22: Figure S13C), harboring at most one repetitive element. They showed a SNP density of up to 11 SNPs/kbp of genome.

84.88% (174 out of 205) of the transferred genes came from a modestly long region between these breakpoints (216 kbp, 4.70% of the *E.coli* genome). We focused on the 29 genes that have no orthologues in the ancestral W recipient genome, reasoning that transfer of these genes may be beneficial for butyric acid adaptation. 25 of these genes fall into two metabolic operons (*ato, rhm)* and the *gtr*, *yfb and yfd* operons. As we discussed above, the *ato* operon is important for butyric acid metabolism [61]. It encodes the short chain fatty acid transporter AtoE, as well as acetate CoA transferase (AtoD-AtoA complex), and acetoacetyl-CoA thiolase (AtoB) [59]. In contrast to the *ato* operon, the *rhm, yfb* and *yfd* operons have not been implicated in butyric acid metabolism (see Additional file 23: Text S3 for details).

### De novo mutations during butyric acid adaptation

In the 30 genomes we analysed, we found 43 mutations (Additional file 24: Excel file S2) in 22 genes (Table 1 and Additional file 25: Table S9) that originated from the ancestral W recipient. Thirteen of these mutations are synonymous. Among the mutated genes are *glpK, rpoB and rpoC* (only *rpoC* showed parallel mutations in one $\operatorname{Re}{\mathrm{c}}_{\mathrm{W}}^{\mathrm{W}}$ and one Rec_W_ population), which may convey general growth benefits in the laboratory environment [67–69]. We also observed parallel mutations in the catabolic regulator genes *crp, cpdA,* and *cpdB* (Additional file 25: Table S9). In addition, parallel mutations occurred in genes involved in acetate production and consumption (e.g *sucA* and *ackA;* Additional file 25: Table S9, Additional file 24: Excel file S2) [62, 70, 71].

## Discussion

To find out whether conjugation-mediated horizontal gene transfer facilitates evolutionary adaptation to novel environments, we subjected multiple replicate populations of *E.coli* to such transfer during laboratory evolution on the novel carbon sources HPA and butyric acid. We did so for different DNA donor-recipient pairs (Fig. 1), to find out whether benefits of horizontal gene transfer might depend on the identity of the donor strains. At the end of laboratory evolution, we analysed whole genome sequencing data of 65 clones isolated from multiple replicate populations to identify potentially adaptive point mutations and horizontally transferred genes. Most genetic changes we were able to detect are likely to have attained a high population frequency, because we sequenced the genomes of at most two clones per population. This high frequency would be the result of selection and/or hitchhiking rather than genetic drift. The reason is that our populations were large, with bottleneck sizes of at least 10^5^ cells, which implies weak genetic drift. Most detectable horizontal transfer events were thus the joint result of the actual transfer event and subsequent natural selection.

In the HPA adaptation experiment, three complementary analyses showed that a K recipient recombining with a different donor experiences a substantial fitness benefit during adaptation on HPA, regardless of the identity of this donor. This benefit is most likely mediated by the transfer of the *hpa* operon, because all sequenced $\operatorname{Re}{\mathrm{c}}_{\mathrm{K}}^{\mathrm{W}}$ and $\operatorname{Re}{\mathrm{c}}_{\mathrm{K}}^{\mathrm{B}}$ clones have acquired this operon. It is fully functional and inducible when HPA is present in the growth medium [55]. That the transfer of the *hpa* operon is adaptively significant is also made likely by the lack of any genes mutated in parallel among $\operatorname{Re}{\mathrm{c}}_{\mathrm{K}}^{\mathrm{W}}$ and $\operatorname{Re}{\mathrm{c}}_{\mathrm{K}}^{\mathrm{B}}$ clones with fast growth in HPA. Moreover, the private mutations and additional horizontally acquired genes of individual clones had no known functions related to HPA or phenylacetate metabolism. Still, the private mutations and co-transferred genes in the $\operatorname{Re}{\mathrm{c}}_{\mathrm{K}}^{\mathrm{W}}$ and $\operatorname{Re}{\mathrm{c}}_{\mathrm{K}}^{\mathrm{B}}$ clones may play a minor role in HPA adaptation or in general adaptation to the laboratory condition. To validate the fitness effects of the *hpa* operon and its potential interactions with other mutations, one could monitor the genotypic and phenotypic evolution of evolving populations over periods of times that are beyond the scope of this study.

Even though all sequenced $\operatorname{Re}{\mathrm{c}}_{\mathrm{K}}^{\mathrm{W}}$ and $\operatorname{Re}{\mathrm{c}}_{\mathrm{K}}^{\mathrm{B}}$ clones contained the *hpa* operon, they also contained multiple other transferred genes and mutations, and differed substantially in their recombination breakpoint regions. Thus, the genetic changes caused by horizontal gene transfer in a recipient genome are very complex, even in this short-term evolution experiment conducted in a simple laboratory environment.

The genetic identity of the donor did not affect final fitness on HPA substantially, but it did affect the incidence of horizontal transfer. Specifically, we observed more transferred genes and recombination breakpoints with the *E.coli* B donor than with the W donor (Additional file 13: Figure S9), possibly indicating a greater number of transfer events, even though the effects of transfer and selection are difficult to disentangle. One might be tempted to explain this pattern with the decreased efficiency of horizontal transfer for genomes of greater sequence distance [72], because the W donor is more distantly related to the K12 recipient than the B donor [22, 51]. However, the differences in nucleotide divergence are very small (1.3% versus 0.8%, Additional file 2: Table S2) and unlikely to solely account for this difference. Alternatively, DNA from the B donor may not have been modified due to engineered mutations in its restriction-modification system [65]. Such DNA might be more easily incorporated into the K12 recipient genome than DNA from the W donor, whose restriction-modification system may not be fully inactivated.

In contrast to HPA, butyric acid provided a more substantial challenge to adapting populations, because multiple such populations became extinct, and the remaining populations required at least 800 generations to acquire viability on butyric acid. Because existing biochemical knowledge suggests that multiple genetic changes may be necessary for growth on butyric acid [61, 62], it is surprising that recombination offered no quantitative fitness advantage in our experiments. That is, the surviving $\operatorname{Re}{\mathrm{c}}_{\mathrm{W}}^{\mathrm{K}}$ and $\operatorname{Re}{\mathrm{c}}_{\mathrm{W}}^{\mathrm{B}}$ populations did not show consistently higher fitness at the end of the experiment.

Only small genomic segments with few genes appeared to have been transferred in the butyric acid experiment in two clones from one $\operatorname{Re}{\mathrm{c}}_{\mathrm{W}}^{\mathrm{K}}$ population (Additional file 22: Figure S13). They include the *ato* operon, which is of known importance for butyric acid metabolism, but did not confer a measurable fitness advantage on populations that acquired it. A possible reason is that *ato* operons transferred from the donor may be subject to xenogenic silencing [73] in the W recipient. Alternatively, their expression and the resulting build-up of high endogenous acyl-CoA levels [60] may induce stress [74], especially on cell membrane integrity [75]. The result may be impaired growth of W recipient cells.

In contrast to this limited role of recombination, and in contrast to HPA-evolved populations, recipient populations evolved on butyric acid showed multiple parallel mutations in the same genes, indicating a selective advantage of such mutations. Among them is the *crp* gene, which showed parallel mutations in seven clones from four (one $\operatorname{Re}{\mathrm{c}}_{\mathrm{W}}^{\mathrm{B}}$ and three $\operatorname{Re}{\mathrm{c}}_{\mathrm{W}}^{\mathrm{W}}$) populations. The c*pdA* gene and its functional paralogue *cpdB* [76, 77] showed mutations in one $\operatorname{Re}{\mathrm{c}}_{\mathrm{W}}^{\mathrm{B}}$ and one $\operatorname{Re}{\mathrm{c}}_{\mathrm{W}}^{\mathrm{W}}$ population (Additional file 25: Table S9). These genes (*crp, cpdA, cpdB*, Additional file 25: Table S9) are involved in cyclic AMP (cAMP) mediated gene expression regulation. cAMP is a nutrient signaling molecule whose concentration decreases upon starvation, which can trigger RpoS-mediated global transcription changes (reviewed in Battesti et al. [78]). The *crp* gene encodes the cAMP receptor protein that monitors cAMP levels, and represses the activation of *rpoS* transcription. Mutations in *crp* can facilitate survival in low pH [79], which is highly relevant for our acidic growth condition, and do so probably by derepressing stress response genes. Conversely, *cpdA* and *cpdB* encode cAMP phosphodiesterases [76, 77] that degrade cAMP. Mutations in the two genes can lead to high cellular cAMP levels [80]. The *fad* operon, which is required for fatty acid degradation, is regulated by cAMP. It can be activated by high concentrations of cAMP, which allows cells to metabolize butyric acid [81]. (We observed no mutations in the coding or promoter regions of FadR, the repressor of the *fadAB* operon [58].) Of note, a high level of cAMP can also relieve the repression of the *ato* operon, thus enhancing butyric acid metabolism [62].

Other parallel mutations occurred in metabolic genes, such as the citrate lyase gene *citF*. Many metabolic enzymes are promiscuous in their ability to catalyze chemical reactions [82, 83], and mutations in such enzymes may also have facilitated butyric acid metabolism. The acetate kinase gene *ackA* showed three different stop-gain mutations in different clones, and may thus have suffered a loss of function. We speculate that these mutations may be adaptive by reducing acetate production in the *E. coli* cytoplasm when butyric acid is present as an energy source. In sum, we identified several de novo mutations whose beneficial effects are suggested by their parallel occurrence in different populations. Some of the affected genes may be involved in butyric acid metabolism, although no one candidate gene has proven causal relevance.

Other evolution experiments have also examined the benefits that horizontal gene transfer of chromosomal genes may confer on microbes evolving in the laboratory [41, 42]. One early study asked whether bacterial conjugation could accelerate adaptation of *E. coli* populations to a constant environment [41]. In this study, conjugation was able to increase the genetic diversity of populations, but showed no substantial effect on adaptation. In another study, three ribosomal protein coding genes of *S. typhimurium* were replaced by orthologues from other species, which resulted in poor fitness due to suboptimal expressions of the foreign genes [42]. Yet another study [43] inserted genomic fragments of bacteria and phages into the *S. typhimurium* chromosome, and found that these horizontally transferred fragments did not improve fitness in glucose minimal medium. These experiments show that the long-term benefits of horizontal gene transfer in wild populations [30, 84] are not necessarily matched by short-term benefits. Our study shows that such benefits can indeed exist, but that they depend strongly on the environment.

One limitation of our experiments is that we were not able to distinguish recombination-induced point mutations from point mutations that occurred independently of recombination. However, it is unlikely that recombination is the major source of the point mutations in our populations. First, the number of point mutations was not much greater in recombining than in non-recombining populations (i.e., populations with and without exposure to a donor), and even smaller in several populations. For example, those populations in the HPA-experiment that experienced the most horizontal gene transfer ($\operatorname{Re}{\mathrm{c}}_{\mathrm{K}}^{\mathrm{B}}$) had about half of the number of *de-novo* mutations as the non-recombining (no donor) Rec_K_ populations (12 vs 20 mutations). Second, the point mutations we observed were generally far from the nearest recombination breakpoint regions, with a shortest distance of 2.9 kbp. Finally, it is also relevant in this regard that the mutation rate of the K12 Hfr donor strain system is no greater than that of our recipient strains [64].

Another limitation is that our *E.coli* recipient cells receive foreign DNA only from other *E.coli* strains, whereas many gene transfer events in nature involve more distantly related organisms [8]. Metagenomic experiments suggest that such distant transfer events can often create novel beneficial phenotypes [85, 86]. Thus, an exciting next step would consist of transferring genes between progressively more distantly related species, to study whether the reduced transfer efficiency [72] is compensated by increased transfer benefits in one or multiple novel environments, and to observe how bacteria rewire these genes to evolve new adaptive traits.

## Conclusion

To our knowledge, ours is the first experimental evolution study that identifies the short-term genomic consequences and benefits of horizontal gene transfer for adaptation to novel nutrients. One of our experiments demonstrated that horizontal gene transfer can help create dramatic phenotypic changes and fitness benefits in an evolving population on the short time scale of laboratory evolution. The other experiment, performed in a different environment, did not reveal such benefits, showing that these benefit may depend strongly on the environment and the foreign DNA donor. The genetic changes underlying the benefits we observed are complex, even on the short time scales of laboratory evolution. Future experiments tracking the adaptation of conjugating populations in more environments may help us understand how the interplay between the environment, the recipient genome, and the donor genome, determine the adaptive benefits of horizontal gene transfer.

## Methods

### Strains

Our evolution experiments rely on several donor and recipient strains of *Escherichia coli* (Additional file 1: Table S1) that are derived from nonpathogenic laboratory strains [51]. We use *E. coli* Rel606, which has an *E. coli* strain B background [87], as the B recipient; *E. coli* BW25113 [88], a laboratory derivative of *E. coli* K12, as the K recipient; a derivative of *E. coli* W [51] that we constructed (Additional file 5: Text S1) in this study as the W recipient. None of the recipient strains harbor antibiotic resistance markers (Additional file 1: Table S1).

As donor strains, we used high-frequency recombination (Hfr) *E. coli* B, K12, and W derivatives capable of donating DNA via conjugation (Additional file 1: Table S1). These strains provide the genetic material for genomic recombination in the recipient genome. Briefly, these strains each contain a chromosomally integrated F plasmid region that harbors bacterial conjugative (*tra*) genes, and three origin of transfer (*OriT*) regions in the genome (Additional file 3: Figure S1, Additional file 5: Text S1 section Cloning). The *tra* genes encode proteins involved in conjugation and the *OriTs* are recognition sequences for DNA transfer initiation in conjugation. We engineered these strains using vectors and plasmids provided by the Kao lab [64]. In these strains, the *traST* genes, which are responsible for mating specificity [89] are inactivated to maximize mating frequency [64].

The donor strains harbor various antibiotics resistant markers because of their construction history (details in Additional file 1: Table S1). In addition, the donor strains are tryptophan auxotrophs, because the conjugative F plasmid was inserted into the *trp* operon. Furthermore, the donor strains also differ in their conjugation efficiency (Additional file 5: Text S1 section Conjugation efficiency assays, Additional file 26: Figure S2). We found that the K donor is able to conjugate efficiently with all recipients (overall conjugation efficiency = 1.80 × 10^− 8^). The B donor conjugated relatively poorly with all recipient strains (overall conjugation efficiency = 4.34 × 10^− 10^), and the W donor conjugated only with the K and W recipient (overall efficiency 2.50 × 10^− 07^) (Additional file 26: Figure S2 and Additional file 27: Table S3).

### Growth media and culture conditions

During experimental evolution, we cultured all *E. coli* strains in Davis minimal broth (DM) (Sigma 93,753) supplemented with 0.002% *w*/*v* thiamine hydrochloride (Sigma-Aldrich T4625). We further prepared our growth media by adding combinations of glucose, glycerol (Sigma G2025), L-Tryptophan (Sigma T8941), 4-Hydroxyphenylacetic acid (Aldrich H50004), and butyric acid (Aldrich B103500) to the DM broth, as detailed below. Butyric acid reduced the pH of the growth medium to pH 6. We archived cultures for further analyses by preparing glycerol stocks (15% final *v*/v) and storing these stocks at − 80°C.

### Identifying carbon sources for experimental evolution

To identify carbon sources suitable for our experiment, we used a combination of flux balance analysis (FBA) and BIOLOG phenotypic microarrays. FBA uses curated models of whole-organism metabolisms to predict growth on specific carbon sources [90]. Our FBA analyses were based on metabolic models iJO1366 for *E. coli* K12 MG1655, iECB_1328 for *E. coli* B REL606 and iWFL_1372 for *E. coli* W [54]. BIOLOG assays determine the extent to which a bacterial population can grow or respire in multiple different growth media [91, 92]. We focused on available data from BIOLOG PM1 and PM2 microarrays [51–53], which test for growth on alternative carbon sources. We started our analysis from carbon sources [54] that FBA predicted to support growth, and then used BIOLOG data to validate the FBA predictions [51–53].

We identified 4-Hydroxyphenylacetate (HPA) as a suitable carbon source for our first experiment, using the B and W strains as donor strains, and the K strain as the recipient strain (Fig. 1). FBA predicted that *E. coli* B REL606 and W but not K12 could grow on HPA [54], a prediction that was confirmed by BIOLOG data [52, 53]. The likely reason is the presence of the *hpa* operon in strains B and W [55].

We identified butyric acid as a second carbon source, using *E. coli* K and B donors to recombine with the W recipient. FBA predicts that *E. coli* K12 and B REL606 but not *E. coli* W can grow on butyric acid, due to the presence of the *ato* operon in the former two strains [61]. However, BIOLOG assays show that only *E. coli* K12 but not B REL606 can grow on butyric acid. Previous studies suggested that butyric acid cannot activate the *ato* operon in wild-type *E. coli* [61, 93] and butyric acid may be toxic to *E. coli* [54]*.* Based on this evidence, we reasoned that adaptation to butyric acid by an *E.coli* W recipient strain may require a combination of horizontal transfer of the *ato* operon and additional beneficial mutations for *ato* operon activation and neutralization of butyric acid toxicity.

Before using these carbon sources in the experiment, we validated the expected growth/no-growth patterns of our strains experimentally, as described in Additional file 5: Text S1 (section Growth characterization of ancestral donors and recipients, Additional file 28: Figure S3). We also performed experiments to ensure that the recipient-donor co-cultures could not grow in the media used for adaptation experiments (Additional file 5: Text S1 section Test for cross-feeding of donor and recipient strains). With these experiments, we also identified the minimum glycerol and maximum alternative carbon source concentrations that support growth of our recipient strains as 0.03% glycerol and 0.17% HPA for the *E. coli* K12 recipient, and 0.035% glycerol and 0.165% butyric acid for the *E. coli* W recipient. These served as the starting conditions of the evolution experiments.

### Daily serial transfer of evolving population

Our HPA-adaptation experiment lasted for 60 days (~ 396 generations), and the butyric acid-adaptation experiment lasted for 175 days (~ 1155 generations). During both experiments, we propagated the evolving populations via 100-fold dilutions into fresh medium, which amounts to approximately 6.6 generations per transfer cycle.

In the HPA (butyric acid) experiment, we gradually replaced glycerol with HPA (butyric acid) to determine whether our populations could evolve to use HPA (butyric acid) as the main carbon source (Additional file 29: Table S5). Specifically, we started with the previously mentioned initial concentration of glycerol and HPA (butyric acid). We gradually decreased the glycerol concentration every 10 days, as shown in Additional file 29: Table S5, to a value of zero during the last ten days of the experiment. Each decrease in glycerol concentration was matched by an increase in the HPA (butyric acid) concentration (Additional file 29: Table S5). We maintained the total concentration of carbon sources at 0.2% in both experiments (Additional file 29: Table S5).

We initialized our experiments with a single overnight pre-culture of the appropriate recipient strain in DM minimal medium supplemented with 0.2% glycerol and 50 μg/ml L-tryptophan. We washed 2 ml of the pre-culture twice in DM broth and transferred 200 μl of the resulting cell suspension to 1800 μl of medium, to a final volume of 2 ml in a well of a 48-well plate (Corning Axygen 12,000-728). We seeded a total of 24 replicate populations in this way (Fig. 1), and incubated the plate at 37°C and 100 rpm in a shaking incubator (Edmund Buhler TH30). We performed 100-fold serial dilution of the evolving culture every 24 h to fresh medium, which introduced a population bottleneck whose severity depended on how fast cells had grown. Its size was approximately equal to 10^6^ cells in the HPA adaptation experiment, and ranged from 10^3^ to 10^6^ cells in the butyric acid adaptation experiment.

To avoid and detect cross-contamination and donor-strain invasion we used several complementary approaches (see Additional file 5: Text S1 section Prevention and detection of cross-contamination among evolving recipient populations). After successful verification that a donor strain had not invaded the population during the previous five days, we added 5 μl of a donor strain culture (see Additional file 5: Text S1 section Donor strain culture preparation) to the appropriate recipient population (Fig. 1) to induce recombination.

### Phenotypic characterization of the evolving populations

After every five days of evolution, prior to the introduction of fresh donor strain, we estimated the fraction of cells that could metabolize the novel nutrients in each evolving population. To this end, we used the following plating assay to estimate the density of cells adapted to the novel nutrient (HPA or butyric acid), and divided this density by the total cell density of the evolving population.

To estimate the density of cells adapted to HPA or butyric acid, we plated 100 μl of 10^2^- and 10^4^-fold diluted population samples on solid DM agar plates supplemented with 0.2% of either nutrient, and counted the number of visible colonies after 48 h of incubation. Likewise, we estimated the *total* cell density in the population by counting the number of visible colonies after 48 h of incubation of 10^4^ and 10^6^-fold diluted samples of an evolving population on solid DM agar plates supplemented with 0.2% glucose. We used glucose instead of glycerol in the latter assay, because our prime objective was to estimate the number of viable cells, and because cells grow faster on glucose.

At the end of the experiment, three and eight replicate populations of the HPA- and the butyric acid-adaption experiments, respectively, went extinct. We confirmed these extinctions by plating undiluted cultures of these populations on DM agar plates supplemented with glucose, and recovered no colonies.

### Phenotypic characterization of the evolved populations at the end of the experiment

We assayed the fitness of the evolved recipient populations via population growth curves measured over 48 h. We did so for a sample of each population, for four individual clones isolated from each population at the end of the experiment, and for the two ancestral recipient strains. We performed each such growth assay in three biological replicates. Thus, in total, we performed 450 growth assays of individual clones: 21 HPA-adapted populations × 4 clones = 84 assays (3 of the initially 24 HPA replicate populations had gone extinct); 16 butyric-acid-adapted populations × 4 clones = 64 clones (8 of the initially 24 butyric acid populations had gone extinct); 84 + 64 +  2 ancestors = 150 clones × 3 replicates = 450 growth assays. To isolate colonies, we spread 100 μl of a 1 × 10^6^-fold diluted sample of an evolved population onto solid DM agar plates supplemented with 0.2% glucose, and incubated for 48 h.

Before measuring fitness, we prepared glycerol stocks from population samples, as well as from isolated clones, from overnight liquid cultures in DM minimal medium supplemented with 0.2% glucose. We also used these glycerol stocks for subsequent genome sequencing of clones.

To measure the fitness of an evolved population (clone) sample in HPA (butyric acid), we first established an overnight culture from glycerol stock in 1 ml of 0.2% glucose-supplemented DM liquid medium, and seeded three replicates from this culture in a TPP flat-bottom 96-well plate by adding 2 μl of the culture to 198 μl fresh DM medium supplemented with 0.2% HPA (butyric acid).

We measured a population’s optical density at 600 nm every 10 min for 48 h on a Tecan Pro200 plate reader. We measured all growth parameters using the R package Growthcurver v0.2.1 [94], and used Growthcurver’s estimates of growth rate in exponential phase as a fitness proxy (Additional file 30: Table S11, Additional file 9: Table S12, Additional file 20: Table S13, Additional file 21: Table S14). We assigned no growth to clones and populations showing an overall optical density change less than 0.05 and a growth trajectories leading to poor fits by Growthcurver. Both ancestral recipients grew so poorly in the novel nutrients that no meaningful growth curves could be measured, which precluded us from measuring the growth rate changes of evolved populations relative to the ancestor.

### Whole genome sequencing of clones

We sequenced the genomes of the ancestral K, B, and W donors, of the ancestral K and W recipients, and of two clones from each of the evolved populations at the end of the evolution experiment (2 × 21 clones from the 4HPA-adaptation experiment and 2 × 16 clones from the butyric acid-adaptation experiment, thus 79 clones in total). We sequenced clones to an average of 99-fold coverage (Additional file 11: Figure S5). To this end, we used a sequencing, alignment, and mutation discovery protocol described in Additional file 5: Text S1.

### Identification of genes likely to be horizontally transferred

When analyzing the sequences of clones from our evolved populations, we used two approaches to identify genes that were probably transferred from a donor to a recipient with a different strain origin (Additional file 10: Figure S14). The first relies on the sequence coverage per gene, and the second relies on polymorphism data. (We excluded from this analysis recipients recombining with donors of the same strain, and recipients not exposed to any donor.)

In the first, coverage-based approach, we computed the fraction of reads covering a given gene that was alignable to either the recipient or donor reference genome (see Additional file 31: Table S15), relative to the total sequence coverage among orthologues of this gene in both genomes. We restricted this analysis to one-to-one orthologues (see Additional file 5: Text S1 section One-to-one orthologues identification) in each donor-recipient pair.

For any one sequenced clone, we aligned reads to reference genomes (Additional file 31: Table S15) of both the appropriate donor and recipient using Bowtie2 in local mapping mode, which reports the best alignment for each read. In this mode, a read originating from a region with 100% sequence identity between the donor and recipient genome would generate one alignment, randomly mapped to one of the genomes. We then computed the number of reads mapped to the donor or the recipient reference genomes (Additional file 1: Table S1) for each gene, using GATK DepthofCoverage [95]. For one-to-one gene orthologues, we then computed the fraction of the coverage attributable to each of the genomes by dividing the sequence coverage of the gene in either genome (donor or recipient) by the total coverage. For example, if 200 reads aligned to the donor’s copy of the gene, and 50 reads to the recipient’s copy, then 100×(200/(200 + 50)) = 80% of reads are of donor origin. For one-to-one orthologues with 100% sequence identity, one would expect that 50% of reads map to the donor, and 50% map to the recipient genome. In contrast, for genes unique to one genome, one would expect that 100% of reads map to that genome.

We assigned a gene as having originated from the donor, if more than 60% of its sequence reads mapped to the donor genome. The number of genes assigned to the donor is not very sensitive to this threshold. The reason is that for 90% of genes assigned to the donor genome more than 90% of reads mapped to the donor (Additional file 32: Figure S6). Increasing the threshold from 60% to 70% (or 80%) only changed the assignments of 1.18% (or 2.82%) of genes. We excluded in total 105 genes present in the ancestral recipient genomes from this analysis, because they appeared to have originated from horizontal gene transfer events that took place before our experiment (Details in Additional file 5: Text S1 section Filtering of genomic regions prone to alignment errors). Overall, we were able to infer the genomic origins of at least 3800 genes for each donor-recipient genome pair (Additional file 33: Table S6, upper part).

Our second approach to identify horizontally transferred DNA used single nucleotide polymorphism data. The main idea is that the sequence of a gene transferred from donor to recipient would differ from its copy in the ancestral recipient, and would be more similar to the ancestral donor copy. In this approach, we first identified candidate genes where the ancestral recipient and donor copies have more than three single nucleotide differences per 1 kbp of gene length. This threshold has been demonstrated as being close to the minimum necessary to assign a unique *E. coli* strain origin to a gene [96]. (See Additional file 2: Table S2 for the overall sequence divergence of our various donor-recipient strain pairs.)

For this analysis, we performed whole genome sequence alignments and genotype variant calling of the ancestral donors, the ancestral recipients, and the evolved clones *jointly* in the workflow described next (Additional file 10: Figure S14).

We simultaneously aligned a clone’s genome sequence reads to the reference genomes (Additional file 31: Table S15) of both the appropriate donor and recipient, using Bowtie2 in multiple mapping mode to report all possible alignments [97]. We eliminated reads uniquely alignable to only one of the two ancestral genomes, and then aligned the rest of the reads separately to either the donor or the recipient genomes using Bowtie2 [97] with default local alignment parameters. These reads are likely to align to homologous regions of both the donor and recipient genomes, but may vary in their sequence from either genome. We then refined the alignment using Picard tools and called all genotypes with minimum mapping quality of 10 using GATK v 3.14 [95], as described previously.

At the end of this procedure, each clone had its genotype called from two alignments, one to the donor and the other to the recipient genome. We then inferred likely codon position within coding regions of the polymorphic sites using snpEff [98]. We focused on codon positions that possessed different alleles between the ancestral donor and recipient; we only further analysed variant sites if the clone’s genotype was consistently called from the two alignments. We assigned a gene to a donor origin if at least three polymorphic nucleotides within 1 kbp had the same genotype as the ancestral donor, but differed from the ancestral recipient (for further details see Additional file 5: Text S1, Identification of horizontally acquired genes using single nucleotide polymorphisms). This approach allowed us to assign a donor origin to between 1594 and 2688 genes, for those experiments where the donor and recipient were different (Additional file 33: Table S6, lower part).

We compared the horizontally transferred genes identified by the two approaches, and found that the alignment-based approach typically identified two times more horizontally transferred genes. The likely reason is that many genes do not show a sufficient number of single-nucleotide polymorphic sites to identify the strain origin, and that many genes are only present in either the donor or the recipient genome. Nonetheless, where both approaches could be used to assign a strain origin, they yielded consistent assignments for more than 99% of genes. Putative horizontally transferred genes also tended to cluster in the donor reference genome (in regions longer than 10 kbp), as one would expect if these genes were indeed transferred horizontally.

### Identifying regions with recombination breakpoints

To find likely regions containing recombination breakpoints for horizontally transferred genomic DNA in each clone, we identified genomic regions where the origin of genes (determined from both the coverage-based and the polymorphism-based approaches) changed from donor to recipient or vice versa. We identified such breakpoint regions for $\operatorname{Re}{\mathrm{c}}_{\mathrm{K}}^{\mathrm{W}},\operatorname{Re}{\mathrm{c}}_{\mathrm{K}}^{\mathrm{B}},\operatorname{Re}{\mathrm{c}}_{\mathrm{W}}^{\mathrm{B}}$ and $\operatorname{Re}{\mathrm{c}}_{\mathrm{W}}^{\mathrm{K}}$ experiments, because these harbored sufficient sequence differences to infer horizontal transfer (Additional file 33: Table S6).

Specifically, we first screened each sequenced clone to identify genes that (i) showed reads alignable to both the donor and recipient genome, and (ii) were adjacent to genes with different strain origin. These genes are likely near a horizontally transferred region or span its boundary. For any one such gene, we used the polymorphism data described in the previous section to gather the strain origin information of at least 30 variant sites within a 2 kbp window upstream and downstream of the gene. For example, for a gene of length 1 kbp, we would survey a total of 5 kbp. The actual length of this window varied depending on the sequence similarity between the donor and the recipient genome in that region. We estimated the likely location of a recombination breakpoint as the region between two adjacent variant sites with different genomic origins, where all surveyed variant sites upstream had one genomic origin (e.g., donor), and all downstream sites had the other genomic origin (e.g. recipient).

### De novo mutation identification

In each sequenced clone, we identified mutations that had occurred during evolutionary adaptation (see Additional file 10: Figure S14). To this end, we aligned all genomic sequence reads from the clone with the recipient and with the donor reference genome (Additional file 1: Table S1) using Bowtie2 [97]. We refined the two resulting alignments and called genotypes (mapping quality > 30) using GATK [95] v3.14 jointly for the ancestral donor, the ancestral recipient, and the evolved clones.

For genes that originated from the recipient genome (as determined from our sequence coverage and polymorphism based analysis), we called genotypes from the alignment to the recipient genome. Conversely, for genes that originated from the donor genome (as determined from our sequence coverage and polymorphism based analysis), we called genotypes from the alignment to the donor genome.

We then focused on nucleotide sites that had no missing genotypes in the ancestral recipient, the ancestral donor, and in the clone itself, and called an allele a de novo mutant if it was different from the genotypes of both the ancestral donor and the ancestral recipient.

## Additional files

Additional file 1: **Table S1.** *E. coli* strains and vectors constructed and used in this project. CGSC: The Coli Genetic Stock Center (DOCX 13 kb)

Additional file 2: **Table S2.** Nucleotide divergence and genomic sequence similarities between pair of *E. coli* reference strains based on Refseq annotation [99], progressive Mauve [100] inference and average nucleotide identity [101]. (DOCX 12 kb)

Additional file 3: **Figure S1.** Schematic diagram of *E. coli* donor strain genotypes. We used three different *E. coli* strains as our DNA donors. Each strain harbored an F plasmid region (blue) integrated into the tryptophan operon. This region contains the *tra* genes encoding the proteins involved in conjugation. The donors are tryptophan auxotrophs, caused by the F plasmid insertion into the *trp* operon. In addition, two origin of transfers (*OriT*s, green) are inserted into each donor genome (at the pseudogenes *mbhA*, *hyfC* genes, or the inactivated genes *hsdRS*, depending on the donor). Thus, including the *OriT* region within the F plasmid, each donor strain has three *OriT* regions. (EPS 376 kb)

Additional file 4: **Figure S4.** The amounts of B, K and W donors in mixed donor-recipient culture decrease rapidly over time. Upper panels: The vertical axes (note the logarithmic scale) show the daily rate of decrease (cfu/ml day; colony forming units per ml of culture per day) in donor density during four consecutive daily serial dilutions, estimated via linear regression [102]. Each colored circle corresponds to data from a donor strain (see color legend), in a mixed culture with either a K recipient and different concentrations of HPA and glycerol (left upper panel), or with a W recipient in different concentrations of butyric acid and glycerol (right upper panel). Lower panels show the nutrient concentrations used (see also Additional file 27: Table S3). We carried out two replicate experiment for each donor-recipient combination per nutrient condition. (EPS 128 kb)

Additional file 5: **Text S1.** Supplementary Methods (DOCX 46 kb)

Additional file 6:**Figure S7.** Density of cells adapted to HPA and total cell density of evolving replicate populations. (A) Total cell density (vertical axis, log scale) of evolving populations as a function of time (horizontal axis), as estimated by plating population samples on glucose-containing plates. (B) Fraction of HPA-adapted cells (Methods), measured every five days (~ 33 generations) during the HPA-adaptation experiment for each replicate population indicated in the color legend.  '$\operatorname{Re}{\mathrm{c}}_{\mathrm{Y}}^{\mathrm{X}}$' denotes populations where an Y recipient recombines with an X donor, and replicate populations for any one such treatment are labeled with numbers in the legend. (C) Concentrations of glycerol (dark yellow) and HPA (cyan) during the experiment. Data from replicate populations we found to be contaminated (Additional file 5: Text S1) are not reported in the Figure. (EPS 416 kb)

Additional file 7:**Figure S8.** Detection of HPA evolved clones recombined with the wrong donor due to contamination during experimental evolution. The first two principal component axes for PCA-based clustering of HPA evolved clones, based on genotypes called successfully across all clones (circles). Asterisks indicate the genomes of ancestral recipients and donors. Note that the B donor is excluded from the plot because it is too far away from the rest of the clones for meaningful data visualisation. Each circle depicts the values of the principal component one (PC1) and two (PC2) of the genotype of an evolved clone (see color legend for the experimental origin ($\operatorname{Re}{\mathrm{c}}_{\mathrm{Y}}^{\mathrm{X}}$) for each clone, where X indicates the donor and Y indicates the recipient). Open circles indicate that recipients had recombined with the appropriate donors. Closed circles indicate apparently inappropriate donors. Specifically, we found six clones (closed circles) from two $\operatorname{Re}{\mathrm{c}}_{\mathrm{K}}^{\mathrm{K}}$ and one Rec_K_ replicate population that clustered with populations exposed to B donors, indicating contamination (Note that the positions of the corresponding six closed circles overlapped extensively, and are thus not all visible). (EPS 73 kb)

Additional file 8:**Table S7.** Statistical tests conducted. df: degrees of freedom, n = number of samples. * Statistical test adopted from [103], methods S1. The Mann-Whitney U-test is computed using the package exactRankTests [104] in R. '$\operatorname{Re}{\mathrm{c}}_{\mathrm{Y}}^{\mathrm{X}}$' denotes a population of Y recipient strains exposed to donor strain X. (DOCX 21 kb)

Additional file 9: **Table S12.** Summary of growth parameters of HPA-evolved clones. We randomly selected four clones from each HPA-evolved population (Methods) and measured the clones’ growth in HPA-supplemented liquid media. The mean and standard deviations of growth rate, carrying capacity and area under the growth curve estimated by Growthcurver v0.2.1 are summarized for three replicate measurements and rounded to three significant digits. (DOCX 18 kb)

Additional file 10: **Figure S14.** Workflow of data analyses. We present the key steps of our data analyses to identify horizontally transferred genes and *de novo* mutations. For evolving populations exposed to a donor of a different strain (top left corner), we used both coverage-based and SNP-based approaches to determine the likely origin of horizontally transferred genes. Then we combined the data from both approaches and used the origin information of transferred genes to identify breakpoint regions and *de novo* mutations. For clones exposed to donors of the same strain or to no donor (top right cornner), we could only use the SNP-based approach to identify horizontally transferred genes. In this case, we used gene origin as inferred by this approach for *de novo* mutation discovery. (EPS 98 kb)

Additional file 11: **Figure S5.** Genome-wide sequence coverage of sequenced clones. Histogram of genome-wide sequence coverage estimated by GATK v3.14 DepthofCoverage [95], for reads of each sequenced clone aligned to the appropriate recipient reference genome. Bin width corresponds to 10-fold coverage. (EPS 46 kb)

Additional file 12: **Table S16.** Summary of data from horizontally transferred genes for each analysed clone in populations where horizontal gene transfer was detected. The table shows the number of unique genes and one-to-one orthologues introduced from the donor, the number of single nucleotide polymorphisms introduced by horizontal gene transfer from the donor genome, the number of *de novo* mutations at recipient background. (DOCX 15 kb)

Additional file 13:**Figure S9.** Putative recombination breakpoints of HPA-evolved clones and the distribution of distances from *OriTs* of horizontally transferred genes. (A) and (C) Circos plots indicating putative recombination breakpoints (Methods) detected in each K recipient clone. The outermost circle (grey) indicates the genomic coordinates (in Mb) relative to the origin of replication (marked as 0) in the *E. coli* K12 genome. Each breakpoint detected is indicated as a radial bar (colored as for the replicate populations in Fig. 2) in one of the concentric inner circles. Breakpoints shared among multiple clones are stacked vertically. Panel A) indicates the genomic location of recombination breakpoints detected in K recipient clones exposed to the B donor ($\operatorname{Re}{\mathrm{c}}_{\mathrm{K}}^{\mathrm{B}}$). Panel C indicates the genomic location of recombination breakpoints detected in K recipient clones exposed to the W donor ($\operatorname{Re}{\mathrm{c}}_{\mathrm{K}}^{\mathrm{W}}$). Note that most breakpoints come in vertically stacked pairs, indicating that the two clones sequenced from the same population usually share recombination breakpoints. (B) and (D) Histograms (bin width of 50 kbp) showing the distribution of distances downstream of the nearest *OriT* of horizontally transferred genes present in the K recipient. We calculated distances for each transferred gene using the known genomic location of the nearest upstream *OriTs*, and did so using the reference *E. coli* REL606 B str. Genome for (B) and the reference *E. coli* W genome for (D). (E) Histogram indicating the number of transposons, prophages, repeat regions, and tandem repeats (see color legend) at 20 kbp intervals along the *E. coli* K12 genome. The top of the panel indicates recombination breakpoints (vertical bars) of the $\operatorname{Re}{\mathrm{c}}_{\mathrm{K}}^{\mathrm{W}}$ (orange) and $\operatorname{Re}{\mathrm{c}}_{\mathrm{K}}^{\mathrm{B}}$ (green) clones. The data suggests that these breakpoints do not fall into regions where these genetic elements have especially high density. (EPS 510 kb)

Additional file 14:**Figure S10.** Rank plot of the number of horizontal gene transfers of genes found among K recipient clones. (A) The number of sequenced recipient clones (y-axis) in which each of 2643 genes appears as transferred from the B donor (x-axis, genes ranked from left to right by descending number of clones) to the K recipient. Data is based on six $\operatorname{Re}{\mathrm{c}}_{\mathrm{K}}^{\mathrm{B}}$ populations and on two sequenced clones per population, i.e., a gene could appear as having been transferred at most 12 times. (B) The number of sequenced recipient clones (y-axis) in which each of 319 genes appears as transferred from the W donor (x-axis, genes ranked from left to right by descending number of clones) to the K recipient. Data is based on five $\operatorname{Re}{\mathrm{c}}_{\mathrm{K}}^{\mathrm{W}}$ populations and on a total of nine sequenced clones, i.e., a gene could appear as having been transferred at most nine times. The 17 genes most frequently transferred from *both* the B and the W donors are highlighted in dark green (A) and dark orange (B). Note that the x-axes in the two panels are not on the same scale, because many fewer genes were transferred in (A) than in (B). (EPS 135 kb)

Additional file 15:**Figure S11.** Percentage of sequenced clones in which *hpa* genes appear horizontally transferred. The bar plot reports the proportion of clones from the $\operatorname{Re}{\mathrm{c}}_{\mathrm{K}}^{\mathrm{W}}$ and $\operatorname{Re}{\mathrm{c}}_{\mathrm{K}}^{\mathrm{B}}$ populations with a horizontally transferred copy of an *hpa* operon gene or an intermediate flanking gene (green: data based on 12 sequenced clones from $\operatorname{Re}{\mathrm{c}}_{\mathrm{K}}^{\mathrm{B}}$ populations that acquired genes from the B donor; orange: data based on nine sequenced clones from $\operatorname{Re}{\mathrm{c}}_{\mathrm{K}}^{\mathrm{W}}$ populations that acquired genes from the W donor) of the *hpa* genes and the genes flanking the *hpa* operon. The schematic diagram at the bottom illustrates genes from the *hpa* operon (pink, gene names above pink arrows, which indicate gene orientation) and two flanking genes (grey) present in the B and the W donor genome (gene lengths not to scale). (EPS 94 kb)

Additional file 16: **Text S2.** Genes transferred to the K recipient from only one but not both donors during evolution on HPA (DOCX 12 kb)

Additional file 17:Excel file S1. List of de novo mutations in coding regions found in sequenced clones isolated from HPA evolved populations. The coordinates of mutations are given in coordinates of (i) the *E. coli* K12 reference genome for mutations in genes from the recipient K12 genome (sheet 1), (ii) the *E. coli* W genome in genes transferred from the *E. coli W* donor in $\operatorname{Re}{\mathrm{c}}_{\mathrm{K}}^{\mathrm{W}}$ clones (sheet 2), and (iii) the *E. coli* B genome in genes transferred from the *E. coli W* donor in $\operatorname{Re}{\mathrm{c}}_{\mathrm{K}}^{\mathrm{B}}$ clones (sheet 3) (In '$\operatorname{Re}{{\mathrm{c}}_{\mathrm{Y}}^{\mathrm{X}}}^{'}$', X refers to the DNA donor identity, and Y refers to the recipient identity). Mutations are annotated according to the amino acid change they cause in the encoded polypeptide sequence. Specifically, the left first three-letter abbreviation denotes the original amino acid, the following number indicates its location, and the right abbreviation denotes the substituted amino acid. ‘fs’ denotes frameshift mutations, ‘del’ denotes a deletion, and ‘*’ denotes a stop-gain mutation. (XLSX 12 kb)

Additional file 18:**Table S8.** Number of clones with a mutant allele in specific genes found in the HPA adapted populations. The coordinates of the start and the end of a gene are listed according to *E. coli* K12 reference genome coordinates. α: Number of variant sites identified within a gene among all HPA evolved clones. Β: Number of clones in a given recombination condition ('$\operatorname{Re}{\mathrm{c}}_{\mathrm{Y}}^{\mathrm{X}}$' , where X is the donor identity, and Y the recipient identity) with the derived allele(s) of the gene. (DOCX 15 kb)

Additional file 19:**Figure S12.** Density of cells adapted to butyric acid and total cell density of the evolving replicate populations. (A) Total cell density (vertical axis, log scale) of evolving populations as a function of time (horizontal axis), as estimated by plating population samples on glucose-containing plates. Missing data indicates that no clones growing on the butyric acid plate were found or the replicate population had gone extinct (replicate populations not showed in the legend). (B) Fraction of butyric acid-adapted cells (Methods), measured every five days (~ 33 generations) during the butyric acid-adaptation experiment for each replicate population indicated in the color legend. '$\operatorname{Re}{\mathrm{c}}_{\mathrm{Y}}^{\mathrm{X}}$' denotes populations where an Y recipient recombines with an X donor, and replicate populations for any one such treatment are labeled with numbers in the legend. (C) Concentrations of glycerol (dark yellow) and butyric acid (pink) during the experiment. (EPS 121 kb)

Additional file 20: **Table S13**. Summary of growth parameters of populations evolved in butyric acid. The table shows mean and standard deviations of growth rate, carrying capacity and area under the growth curve estimated by Growthcurver v0.2.1 at the end of the butyric acid-adaptation experiment based on three replicate measurements of growth in butyric acid-supplemented liquid medium and rounded to three significant digits. (DOCX 13 kb)

Additional file 21: **Table S14.** Summary of growth parameters of butyric acid-evolved clones. We randomly selected four clones from each butyric acid-evolved population (Methods) and measured the clones’ growth in butyric acid-supplemented liquid media. The mean and standard deviations of growth rate, carrying capacity and area under the growth curve estimated by Growthcurver v0.2.1 are summarized for three replicate measurements and rounded to three significant figures. (DOCX 17 kb)

Additional file 22:**Figure S13.** Putative recombination breakpoints of butyric acid-evolved clones and the distribution of distances from *OriTs* of horizontally transferred genes. (A) Circos plots indicating putative recombination breakpoints (Methods) detected in eight out of ten recipient clones exposed to the K donor. The outermost circle (grey) indicates the genomic coordinates (in Mb) relative to the origin of replication (marked as 0) in the *E. coli* W genome. Each breakpoint detected is indicated as a radial bar (colored as for the replicate populations in Fig. 3) in one of the concentric inner circles. Breakpoints shared among multiple clones are stacked vertically. Panel (A) indicates the genomic location of recombination breakpoints detected in W recipient clones exposed to the K donor ($\operatorname{Re}{\mathrm{c}}_{\mathrm{W}}^{\mathrm{K}}$). Location of genes (*yejA* and *yfdE*) spanning or adjacent to the breakpoint regions are marked. (B) Histograms (bin-width of 50 kbp) showing the distribution of distances downstream of the nearest *OriT* of horizontally transferred genes present in the W recipient. We calculated distances for each transferred gene using the known genomic location of the nearest upstream *OriTs*, and did so using the reference *E. coli* W genome. (C) Histogram indicating the number of transposons, prophages, repeat regions, and tandem repeats (see color legend) at 20 kbp intervals along the *E. coli* W genome. The top of the panel indicates recombination breakpoints (vertical bars) of the $\operatorname{Re}{\mathrm{c}}_{\mathrm{W}}^{\mathrm{K}}$ (blue) clones. The data suggests that these breakpoints do not fall into regions where these genetic elements have especially high density. (EPS 185 kb)

Additional file 23: Text S3. Potentially adaptive gene transfers from the K donor to the W recipient clones during evolution on butyric acid (DOCX 15 kb)

Additional file 24:Excel file S2. List of de novo mutations in coding regions found in sequenced clones isolated from butyric acid evolved populations. The coordinates of mutations are given in *E. coli* W reference genome coordinates (sheet 1) (In '$\operatorname{Re}{\mathrm{c}}_{\mathrm{Y}}^{\mathrm{X}}$', X refers to the DNA donor identity, and Y refers to the recipient identity). Mutations are annotated according to the amino acid change they cause in the encoded polypeptide sequence. Specifically, the left first three-letter abbreviation denotes the original amino acid, the following number indicates its location, and the right abbreviation denotes the substituted amino acid. ‘fs’ denotes frameshift mutations, ‘del’ denotes a deletion, and ‘*’ denotes a stop-gain mutation. We did not observed any mutations in transferred genes from the B donors in the $\operatorname{Re}{\mathrm{c}}_{\mathrm{W}}^{\mathrm{B}}$ or K donors in the $\operatorname{Re}{\mathrm{c}}_{\mathrm{W}}^{\mathrm{K}}$ clones. (XLSX 12 kb)

Additional file 25:**Table S9.** Number of clones with a mutant allele in specific genes found in the butyric acid-adapted populations. The coordinates of the start and the end of a gene are listed in coordinates of the *E. coli* W reference genome. α: Number of the of a gene variant sites identified within the given gene among all butyric acid-evolved clones. Β: Number of clones in a given recombination condition (‘$\operatorname{Re}{\mathrm{c}}_{\mathrm{Y}}^{\mathrm{X}}$’ X: the donor identity; Y: the recipient identity) that harbor derived allele(s) of the given gene. (DOCX 15 kb)

Additional file 26: **Figure S2.** Conjugation efficiencies of donor and recipient strains. Each box-whisker plot reports the conjugation efficiency of a combination of donor (right vertical axis) and recipient (horizontal axis) in units of the number of transconjugants per donor cell present in the culture (left vertical axis, logarithmic scale). Each plot is based on three independent replicates, each consisting of two individual experiments for each donor-recipient pair. Note that the W donor does not conjugate successfully with the B recipient. Each box plot reports the median conjugation efficiency (center bar), the first and third quartiles (top and bottom bar), and the range of 95% interval of the data (whiskers). (EPS 82 kb)

Additional file 27: **Table S3.** Summary of strains used in the conjugation assay, their selective markers, and the locations of these markers in the genome. (DOCX 12 kb)

Additional file 28: **Figure S3.** No growth of donors and recipients in media used for experimental evolution. Mean growth (circles (donor) and squares (recipients); bars extend to one standard deviation above or below mean; measured as the groeth rate) after 48-h of potential donors (top panels) and recipients (bottom panels, Additional file 1: Table S1) in four growth conditions (0.2% HPA, 0.2% butyric, 0.2% glucose, 0.2% glucose and 50 μg/ml tryptophan) for K12 (blue), B (green) and W (orange) strain background. We only measured and report growth of the recipient strain (K) and the three donor (Fig. 1) used in the HPA-adaptation experiment on 0.2% HPA. Likewise, we only measured growth of the recipient strain (W) and the three donor used (Fig. 1) for the butyric acid experiment in 0.2% butyric acid. The horizontal line indicates zero growth. A summary of ancestral growth parameters is given in Additional file 34: Table S10. (EPS 51 kb)

Additional file 29: **Table S5.** Nutrient concentrations during the evolution experiments. (DOCX 11 kb)

Additional file 30: **Table S11.** Summary of growth parameters of populations evolved in HPA. The table shows mean and standard deviations of growth rate, carrying capacity and area under the growth curve estimated by Growthcurver v0.2.1 at the end of the HPA-adaptation experiment based on three replicate measurements of growth in HPA-supplemented liquid medium and rounded to three significant digits. (DOCX 14 kb)

Additional file 31: **Table S15.** Summary of alignment strategies. The table lists the reference genome(s) we used in whole-genome sequence alignment using Bowtie2 in each of several analyses (columns) described in the methods, and for each adaptation experiment (rows). (DOCX 15 kb)

Additional file 32: **Figure S6.** Proportion of sequence reads per gene that map to the donor genome. In our data, we found in total 15,962 genes (in genomes of 65 clones) had any reads mapped to the donor genome. We plotted the number of genes (bar height, see y-axis) having a proportion of reads mapped to the orthologous copy of the donor genome (see colour scheme) (at 10% interval). The vertical line marks the 60% threshold we used to determine whether a gene originated from the donor or not. Among 15,962 genes that had any reads mapping to the donor genome, 96.42% (15391) of genes passed the 60% threshold (vertical line), 95.28% (15208) passed the 70% threshold, 93.81% (14975) passed the 80% threshold, and 90.98% (14522) passed the 90% threshold. The data indicates that our assignment of transferred genes to the donor genome is not highly sensitive to the 60% threshold we used. (EPS 76 kb)

Additional file 33: **Table S6.** Number of genes suitable to infer horizontally transferred genes for every combination of donor (columns) and recipient (rows) in both the HPA and butyric acid adaptation experiments. We note that ancestral recipient and donor strains of the same backgrounds may differ in DNA sequence at a small number of nucleotide sites as a result of their strain construction. Thus the number of genes suitable for horizontal gene transfer inference are specific for a donor-recipient combination. ND: not determined. (DOCX 12 kb)

Additional file 34: **Table S10.** Summary of growth parameters of ancestral strains. Growth parameters of the ancestral *E. coli* K12, B and W donor and recipient measured in liquid media supplemented with HPA, butyric acid, glucose and glucose with tryptophan. Specifically, the table shows mean and standard deviations of growth rate, carrying capacity and area under the growth curve of each ancestor from three replicate measurements and rounded to three significant digits. (DOCX 14 kb)

Additional file 35: **Table S4.** Nutrient concentrations tested for cross-feeding experiments. (DOCX 11 kb)

## Abbreviations

HGT: Horizontal gene transfer
HPA: 4-Hydroxyphenylacetic acid
