# Supplementary material for: Assessing the benefits of horizontal gene transfer by laboratory evolution and genome sequencing
Source: BMC Evol Biol. 2018 Apr 19;18:54. doi: 10.1186/s12862-018-1164-7 (PMC5909237; doi:10.1186/s12862-018-1164-7)
Supplement: Supplementary file 1 — Table S1. E. coli strains and vectors constructed and used in this project. CGSC: The Coli Genetic Stock Center (DOCX 13 kb) [file 12862_2018_1164_MOESM1_ESM.docx]

| Name assigned in this project | *E. coli* strain | | Reference genome | Source | | | Description | Engineering in this work | |
| --- | --- | --- | --- | --- | --- | --- | --- | --- | --- |
| B  recipient | REL606 | | NC_012967.1 | CGSC 12149 | | | tsx-467(Am), *araA*230, *lon^-^*, *rpsL*227(strR), *hsdR-* | - | |
| K recipient | BW25113 | | NC_000913.3 | CGSC 7636 | | | Δ(araD-B 567), Δ*lacZ*4787(::rrnB-3),  Δ(*rhaD-B*)568,  hsdR514 | - | |
| W recipient | W Δ*hsdR-S, ΔmrcB-C* | | NC_017635.1 | This work | | | W | Δ*hsdR-S, ΔmrcB-C* | |
| B donor | REL606 2×O*riT* Hfr Δ | | NC_012967.1 | This work | | | REL606 2×*OriT* | *trp::*F[Δ*traST* :: kan](genR) | |
| K donor | BW25113 2×O*riT* Hfr Δ | | NC_000913.3 | Winkler et. al. 2012 [64] | | | BW25113 Δ*mbh*A::[oriT cat], Δ*hyfC:*:[oriT kan], *trp::*F[Δ*traST* :: kan](genR) | - | |
| W donor | W 2×O*riT* Hfr Δ | | NC_017635.1 | This work | | | W 2×*OriT* | *trp::*F[Δ*traST* :: kan](genR) | |
| - | CAG31031 *traST* | | - | Winkler et. al. 2012 [64] | | | BW25113 *trp::*F[Δ*traST* :: kan](genR), ycdN::[oriT tetAR] | *-* | |
| - | REL606 2×O*riT* | | - | Winkler unpublished | | | Δ*mbh*A::[oriT cat], Δ*hyfC:*:[oriT kan] | - | |
| - | W | | - | Archer et al 2011 [51] | | | - | - | |
| - | W 2×*OriT* | | - | This work | | | W Δ*hsdR-S, ΔmrcB-C* | Δ*hsdR-S*::[oriT kan], Δ*hyfC*::[oriT cat] | |
| Plasmids and vectors | | | | | | | | | |
| PKD46 plasmid | - | - | | | CGSC 7739 | - | | | - |
| PKD4::oriT plsamid | - | - | | | Winkler 2012 [64] | - | | | - |
| PKD3 | - | - | | | CGSC 7631 | - | | | - |
| PKD3::oriT | - | - | | | Winkler 2012 [64] | - | | | - |
| PCP20 | - | - | | | CGSC 7629 | - | | | - |
