# Supplementary material for: Assessing the benefits of horizontal gene transfer by laboratory evolution and genome sequencing
Source: BMC Evol Biol. 2018 Apr 19;18:54. doi: 10.1186/s12862-018-1164-7 (PMC5909237; doi:10.1186/s12862-018-1164-7)
Supplement: Supplementary file 2 — Table S2. Nucleotide divergence and genomic sequence similarities between pair of E. coli reference strains based on Refseq annotation [99], progressive Mauve [100] inference and average nucleotide identity [101]. (DOCX 12 kb) [file 12862_2018_1164_MOESM2_ESM.docx]

| Strain 1 | Strain 2 | Single nucleotide differences per kilo-base pair of gene othologues | Number of one-to-one gene ortholgoues | Number of genes only present in Strain 1 | Number of genes only present in Strain 2 | Average nucleotide identity (%) |
| --- | --- | --- | --- | --- | --- | --- |
| *E. coli* K12 | *E. coli* B | 8 | 3917 | 577 | 367 | 99.04 |
| *E. coli* K12 | *E. coli* W | 13 | 3921 | 573 | 817 | 98.55 |
| *E. coli* W | *E. coli* B | 14 | 3830 | 906 | 454 | 98.49 |
