# Supplementary material for: Assessing the benefits of horizontal gene transfer by laboratory evolution and genome sequencing
Source: BMC Evol Biol. 2018 Apr 19;18:54. doi: 10.1186/s12862-018-1164-7 (PMC5909237; doi:10.1186/s12862-018-1164-7)
Supplement: Supplementary file 5 — Text S1. Supplementary Methods (DOCX 46 kb) [file 12862_2018_1164_MOESM5_ESM.docx]

**Text S1: Supplementary methods**

Cloning

We performed all cloning experiments in Luria-Bertani (LB) broth (Becton, Dickinson and company, Difco 244610), in LB solid agar and in Hanahan's Borth (Sigma H8021) supplemented with 20 mM glucose (Sigma G7021). We incubated our strains at 37°C unless otherwise specified. We used the following antibiotics in our experiment: chloramphenicol (Sigma C0378), gentamycin sulfate (AppliChem A14920010), kanamycin sulfate (Sigma K1377), nalidixic acid (Simga-Aldrich N8878), and rifampicin (Sigma R3501). See below for specific concentration(s) of antibiotics used in individual experiments.

We construct the W recipient by inactivating the *hsdRS* and *mrcBC* genes in *E.coli W* using the λ red-mediated gene replacement system [1]. These genes are involved in the type III restriction-modification system, which degrades foreign DNA [2]. *hsdRS* encodes a restriction endonuclease, which cleaves unmethylated DNA sequences; *mrcBC* encodes a DNA methyltransferase, which methylates the host genome, protecting it from restriction [2].

All donor strains contain a chromosomally integrated F plasmid region that harbors bacterial conjugative (*tra*) genes. In these strains, the *traST* genes, which are responsible for mating specificity [3] are inactivated to maximize mating frequency [4]. The F plasmid is integrated into the *trp* operon, leading to tryptophan auxotrophy in all three donor strains. In addition, these strains contain not just one origin of transfer (*OriT*) region (inside the F plasmid integrate) but three of these regions, two of which are placed outside the integrate (Figure S1). *OriT* sequences are recognition sequences for the conjugative proteins binding to and initiating DNA transfer during conjugation (Table S1). In the *E. coli* K and B donor, these two *OriT* sequences are inserted into the pseudogenes *mbhA* and *hyfC*. We obtained the K donor directly from the Kao lab [4], (Table S1), and engineered the additional two *oriT* regions into *mbhA* and *hyfC* ourselves to construct the B donor. Because *E. coli* W lacks the *mbhA* orthologue, we inserted the two *OriT*s into *hsdRS* and *hyfC*. We note that in all three donor strains the three *OriT* regions occur at widely spaced genomic locations to maximize the diversity of transferred DNA (Figure S1).

Conjugation efficiency assays

We tested the conjugation efficiency of each of our donor strains with each recipient strain. The conjugation assays we used determine how frequently a donor transfers a specific antibiotic resistance marker to the recipient. These assays involved the four antibiotics chloramphenicol, gentamycin, nalidixic acid, and rifampicin. The genes encoding resistance to them are located in different genomic regions and are at least five minutes (~ 400 kbp) apart from each other (Table S3). As a result of their construction, the donor strains are already resistant to chloramphenicol and gentamycin. However, they are susceptible to nalidixic acid and rifampicin. For the purpose of the conjugation assays, we thus wished to create K, B, and W recipient strains that are resistant to nalidixic acid and to rifampicin, taking advantage of the fact that these resistances can emerge spontaneously and are associated with mutations in the genes *gyrB* and *rpoB*, respectively [5,6]. Mutations in *gyrB* cause nalidixic acid resistance by altering the interaction of the two gyrase subunits B and A (encoded by *gyrA*) [7]. *rpoB* mutations cause rifampicin resistance by reducing the antibiotic’s affinity to the ß-subunit of RNA polymerase [8].

To isolate spontaneous mutants resistant to both rifampicin and nalidixic acid, we set up an overnight culture of a recipient strain in 1 ml of LB liquid medium and plated 100 μl of this culture onto a LB solid agar plate supplemented with 10 μg/ml of nalidixic acid. We incubated the plate for 48 hours, and then streaked up to 20 of the resulting colonies onto an LB plate supplemented with 30 μg/ml nalidixic acid and 10 μg/ml rifampicin. We repeated this procedure (isolation of colonies and incubation on doubly-selective plate for 48 hours), and prepared glycerol stocks of the resulting doubly resistant mutants from overnight cultures in LB liquid medium supplemented with 30 μg/ml nalidixic acid and 100 μg/ml rifampicin.

We reported the conjugation efficiency estimate that resulted from these assays as the transconjugant density divided by the donor cell density, and averaged this fraction over replicates [9] (Figure S2). We further averaged the conjugation efficiencies on both selective plates for each donor-recipient combination.

To conduct the conjugation assay for each pair of donor-recipient strain, we established separate overnight cultures of the donor strains and the (antibiotic resistant) recipients in 5 ml LB liquid medium supplemented with 1) 30 μg/ml gentamycin for the donor, and 2) 30 μg/ml nalidixic acid and 100 μg/ml rifampicin for the recipient. We washed the overnight cultures three times in DM broth and resuspended them in DM broth. Then we mixed the resuspended culture of the donor and recipient strain by adding 20 μl of donor cell suspension to 80 μl of recipient cell suspension. Subsequently, we dispensed 10 μl of the donor-recipient mixture in 1 ml of fresh LB and incubated for 24 hours. As negative controls, we also dispensed 10 μl of donor or recipient suspension to 1 ml of fresh LB and incubated for 24 hours as negative controls. Then we plated 100 μl of the undiluted, 100-fold and 10^4^-fold diluted culture on LB agar plates supplemented with two combinations of antibiotics: 1) gentamycin, nalidixic acid and rifampicin, and 2) chloramphenicol, nalidixic acid and rifampicin. In addition, to estimate the cell density of the donor and the recipient cells before conjugation, we plated 100 μl of 10^4^ and 10^6^-fold diluted negative controls of the donor and the recipient culture on nonselective LB agar plates, incubated for 24 hours, and counted visible colonies.

Only transconjugants are expected to grow on the selective plates, because doing so requires having obtained either the gentamycin or chloramphenicol resistant genes via recombination. We estimated the cell density of the transconjugants by counting visible colonies on these plates after 24 hours of incubation. The negative controls showed no visible colonies.

We conducted each of these assays in triplicate (three biological replicates), and also performed two technical replicates for each donor-recipient combination, to minimize the effect of cell density variation in overnight cultures of donor and recipients on the number of transconjugants. In total, we performed 108 assays (9 donor-recipient combinations x 2 selective conditions x 2 technical replicates x 3 biological replicates).

These assays showed that both K and B donors are capable of conjugating with all recipients, while the W donor could only conjugate with the K and the W recipient (Figure S2). More specifically, the K donor conjugated with the K, B and W recipients at averaged efficiencies of 8.52 x 10^-8^, 1.19 x 10^-8^ and 1.53 x 10^-8^ transconjugants per donor cell, the B donor conjugated with the K, B and W recipient at averaged efficiencies of 3.25 x 10^-9^, 2.56 x 10^-10^, and 3.84 x 10^-10^, and the W donor conjugated with the K and W recipient at averaged efficiencies of 2.86 x 10^-6^ and 3.86 x 10^-7^ transconjugants per donor cell. We note that these are conservative estimates of the conjugation frequency because the selective markers were at least 200 kpb away from the upstream *OriT* sequence (Table S2 and Figure S1).

Growth characterization of ancestral donors and recipients

Before beginning our evolution experiments, we measured growth of our recipient and donor strains in HPA, in butyric acid, as well as in the presence and absence of tryptophan. This was necessary to ensure that the ancestral recipient strains cannot grow on HPA or butyric acid, and that the donor strains are not able to grow without tryptophan (and are thus not likely to invade the recipient population).

To do so, we established overnight pre-cultures of the donor and recipient strains (Table S1) in 5 ml of DM broth supplemented with 0.2% glucose and 50 μg/ml tryptophan. We washed 1 ml of the overnight culture twice in DM liquid medium, and resuspended the culture to a final volume of 1 ml in DM medium. We added 2μl of the resuspended culture to 198μl of DM medium supplemented with 0.2% of HPA or butyric acid. As a control and to verify the tryptophan auxotrophy of the donor strains, we resuspended the same quantity of culture in DM medium with 0.2% glucose, in the presence or absence of 50 μg/ml tryptophan. We conducted these experiments in a flat-bottom TPP 96-well microplate (Sigma Z797910), and measured the optical density at 600 nm on a Tecan Pro200 plate reader every 10 minutes for 48 hours as a proxy of population density. We then estimated growth parameters from the resultant growth curves using the R package growthcurver v0.2.1 [10]. We used the growth rate as our primary growth proxy. The growth parameter estimates are summarized in Table S10.

As expected, donor strains were not able to grow on glucose alone, but only with tryptophan supplementation (Figure S3), thus confirming the insertion of the F plasmid. In contrast, all recipient strains grew in glucose, with or without tryptophan, and generally grew more poorly on glucose and tryptophan than on glucose alone.

In addition, our experiments confirmed that the donor strains could not grow on HPA alone either, due to their tryptophan auxotrophy, and that the K recipient strain could not grow on HPA (Figure S3 bottom left panel), consistent with published BIOLOG data [11,12]. For the butyric acid adaptation experiment, none of the donor strains could grow on medium containing only butyric acid, owing to their tryptophan auxotrophy. Furthermore, as predicted by previous BIOLOG data and growth assays [13], the W recipient strain could not grow on butyric acid either (Figure S3). In sum, the donor and recipient strains displayed the necessary growth characteristics for our HPA and butyric acid experiments.

**Test for cross-feeding of donor and recipient strains**

We confirmed that the donor strains were not able to grow in the medium to be used for experimental evolution. We also wanted to confirm that our donor strains were not able to grow (and thus co-evolve) with the recipient populations, for example, because the recipient might produce excess tryptophan that they can use. To this end, we conducted several pilot evolutionary experiments, each of which lasted four days (approximately 26.4 generations). In these experiments, we used various combinations of carbon sources (Table S4), and monitored the survival of the donor strains on them. Specifically, we first established separate overnight cultures of the donor and recipient strains in 2 ml of DM liquid medium supplemented with 0.2% glycerol and 50 μg/ml tryptophan. We washed 1 ml of each overnight culture 2 times in DM liquid medium, resuspended it in 1 ml of DM liquid medium, and mixed the donor and recipient strains at a ratio of 1:4 v/v by adding 20 μl of donor to 80 μl of recipient suspension. We then transferred 10 μl of the donor-recipient mixture to 990 μl of growth medium (Table S4) to seed the experiment.

For each donor-recipient and carbon source combination (3 donors x 2 recipients x 4 conditions=24 conditions) (Table S4), we performed two replicate experiments, leading to 48 replicate populations. We incubated these populations in 12 wells of 24 round-bottom well plates (Aldrich WHA77015102), arranged in a checkerboard pattern to reduce the possibility of cross-contamination (Edmund Buhler TH30 shaking incubator at 100 rpm and 37°C). We estimated each donor’s cell density in the culture after 24 hours of growth, before performing the daily serial transfer (at 100-fold dilution) of the evolving replicate population. To estimate the donor's cell density (as the number of colony forming units/ml) we plated 100 μl of an 10^4^ and a 10^6^-fold dilution of the evolving culture on LB solid agar supplemented with 25 μg/ml chloramphenicol and 30 μg/ml gentamycin, and counted the number of visible colonies formed after 24 hours of incubation. We estimated the rate of decrease of the donor strain in each population per day using linear regression in R [14] (Figure S4).

We found that the donor strains' cell density decreased by at least 1000 fold at every measurement time point where glycerol was still present in the growth medium (Figure S4). In growth media lacking glycerol, the donor strain’s cell density decreased even more rapidly (10^4^ - 10^6^ cfu/ml/day). These findings suggest that the donor strains do not persist indefinitely, but are eventually diluted out of the evolving population.

**Prevention and detection of cross-contamination among evolving recipient populations**

We implemented three tests for cross-contamination of the evolving recipient populations during experimental evolution. First, we arranged them on the plate in a checker-board pattern (separated by blank wells) to reduce the possibility of cross-contamination. We nonetheless observed two cases of cross-contamination during the experiment. In both cases, we resumed the experiment from the previous day-culture, which we had stored at 4 °C.

Second, we checked for donor invasions into the recipient cell population. Every five days, we sampled and spread 100 μl of the evolving culture on LB solid agar supplemented with gentamycin, to which donor cells with the F plasmid integrate are resistant (Table S1). We counted the number of visible colonies to estimate the population density of the donor strain within the recipient population. We only performed the next round of donor strain introduction (described below) if the donor cell density within each recipient population was lower than 1000 cfu/ml.

In addition, we performed multiplex PCR using strain-specific primers [15] to validate that the evolving recipient populations were not overtaken by the donor strains. We found that all K recipient populations adapting to HPA showed an *E. coli* K12 specific PCR band (1600 bp) [15] as the brightest band. Thus, as expected, these populations consisted mainly of K recipient cells. Similarly, we found that all W recipient populations adapting to butyric acid showed an *E. coli* W specific band (500 bp) [15], indicating that the donor strains had not invaded these recipient populations either.

Using these methods, we identified two likely contamination incidents where empty wells in a plate had shown bacterial growth after 24-hour incubation before the daily serial transfer, and resumed the experiments using populations from the previous day stored at 4° C.

Despite these precautions, whole genome sequence data of evolved clones suggested that three of our HPA-evolved replicates might have been recombining with the wrong donor. This was revealed by a principal component analysis (Methods) of the genotypes of all HPA evolved clones determined from alignments to the *E. coli* K12 reference genome. For this analysis, we used genotypes based on high confidence polymorphic sites (genotype quality > 80), which are mainly located in gene orthologues present in the genomes of all three (K12, B and W) strains. Additionally, we only used alleles located within protein coding regions in the analysis, which is based on a total of 547 synonymous and non-synonymous polymorphic sites.

The analysis showed that HPA-evolved clones split into three main groups, most of which clustered with the expected donor (B, W, or K; S8 Fig.). However, six clones from three replicate populations clustered unexpected with the clones exposed to the B donor, even though they were supposedly only exposed to K donor (or to no donor at all, S8 Fig.). We excluded these likely contaminants from further analysis.

An analogous principal component analysis based on 566 polymorphic sites in butyric acid-evolved clones (not shown) revealed no signs of contamination.

**Donor strain culture preparation**

We had prepared a glycerol stock of each donor strain at the beginning of the experiment in the following way. We established a 20 ml overnight culture of the donor strain in DM liquid medium supplemented with 0.2% glucose and 30 μg/ml gentamycin. We washed this culture three times and resuspended it in 20 ml of DM broth to remove the antibiotics. Then we prepared 100 glycerol stocks from the donor strain culture by adding 100 μl of the culture to 50 μl of 50% glycerol.

To obtain a donor sample for addition to the recipient culture, we thawed one of the glycerol stocks of the donor strain, washed the cell suspension in 1 ml of DM liquid medium, and resuspended the cells in 100 μl of DM liquid medium. We then added 5 μl of the appropriate donor strain, according to the recombination condition (Fig 1), along with 20 μl of the recipient population to fresh growth medium during the serial transfer. We note that this practice of obtaining donor samples from a single ancestral glycerol stock ensures genetic uniformity of the donor samples.

**Library preparation for sequencing**

For each clone, we isolated genomic DNA from 1 ml of overnight culture using the QiAampDNA Mini kit (Qiagen 51304). We used the NEBNext Ultra DNA library preparation kit for Illumina (NEB E7370L), and NEBNext Multiplex Oligos for Illumina (Dual Index Primers Set 1; NEB E7600S), preparing indexed libraries according to Manual E7370. We sequenced whole genome DNA fragments of length between 500 to 800 bp using the Agencourt AMPure XP system (Beckman Coulter E6260). We pooled all indexed libraries and sequenced 2 × 150 bp paired-end reads using the Illumina NextSeq 500/550 sequencer at the Functional Genomic Center Zurich. We sequenced the evolved clones to an average 99.9-fold genome-wide sequence coverage and at minimum 25-fold coverage (Figure S5). We further eliminated one $\mathrm{Re}c_{K}^{W}$ clone due to library failure and two $\mathrm{Re}c_{W}^{B}$ clones due to indexing errors.

### **Whole genome sequence data alignment and mutation discovery**

We used Trimmomatic 0.27 [16] to remove specific Illumina sequencing adaptors, low quality read ends (base quality lower than 10) and trimmed sequence reads shorter than 40 bp. We aligned the reads against the appropriate reference genome using Bowtie2 [17] in local alignment mode, which specifically performs alignment using part(s) of a read most highly similar to the reference genome and detects small-scale local mismatches. We used combinations of *E. coli* K12 reference genome NC_000913.3 [18], *E.coli* B reference genome NC_012967.1 and *E. coli* W reference genome NC_017635.1 [19] in the alignments for each group of evolved populations (see Table S16).

We refined all alignments around indels using Picard tools (http://picard.sourceforge.net.), GATK v3.14 RealignerTargetCreator and IndelRealigner [20]. After alignment, we called all genotypes at sites with point mutations and small insertion/deletions (indels < 5bp) using GATK v3.14 HaplotypeCaller in haploid mode [21]. We called genotypes separately for each group of clones that had been exposed to different donors. We used SnpEff [22] to estimate the likely phenotypic effect of the discovered mutations. We eliminated all SNPs identified in genomic regions prone to alignment errors due to repeats and duplicated genes prior to further analyses.

Filtering of genomic regions prone to alignment errors

We identified regions prone to alignment errors by Bowtie2 [80], and eliminated SNPs and indels located within these regions from further analyses. Specifically, we identified these regions by the enrichment of low mapping-quality alignments and partially alignable reads. We used both of these criteria because they reflect two different types of alignment artefacts: Genomic regions with repetitive elements or genes with multiple paralogues can lead to alignment artefacts with low mapping quality, whereas boundaries of structural variants and intergenic regions of duplicated genes can lead to partially alignable reads. Here are the specific filtering steps we took.

First, we identified genes with poor mapping quality (mapping quality < 5, mapping accuracy < 70%) in the ancestral recipient genomes. For 95% of the genes of the ancestral K (W) recipient, at least 86.74% (75.87%) of reads aligned well to *the E. coli* K12 (W) reference genome (mapping quality ≥ 30). We also eliminated 119 *E. coli* K12 genes and 179 E. coli W genes from further analyses, because these genes were enriched in likely alignment artefacts (> 60% of reads aligned to these genes had mapping quality ≤ 10, equivalent to lower than 90% accuracy).

Next, we focused on reads that are only partially alignable to the reference genome (soft-clipped reads), and computed the genome-wide distribution of soft-clipped base pairs (bps) in reads aligned to the reference genome as follows. We identified reads containing soft-clipped base pairs, and recorded the positions of soft-clipped base pairs relative to the first or last mappable base pair (depending on whether the clipping occurred at the start or end of the read). For example, if only the first 50 base pairs of a 150 bp long hypothetical read mapped to position 1000 – 1050 of the reference genome, we considered the rest of the read (100 base pairs) as soft-clipped, and assigned positions 1051 - 1150 as soft-clipped base pairs. We did this for all soft-clipped reads, and then computed the mean number of soft-clipped base pairs, normalized by the number of mappable base pairs, for each base pair location of the recipient genome. We then computed the average proportion of soft-clipped base pairs for each gene. This analysis showed that 95% of genes in the ancestral K recipient had fewer than 0.013 soft-clipped base pairs per mappable base pair (soft-clipped base pairs/averaged mappable base pair per gene=2.99/239.5). Similarly, in the W recipient 95% of genes had fewer than 0.017 soft-clipped base pairs per mappable base pair (soft-clipped/gene coverage=3.01/182). We eliminated the 5% of genes (184 in the *E. coli* K12 and 247 in the *E. coli* W genome) with the highest number of soft-clipped base pairs per mappable base pair from further analyses.

In sum, we eliminated 272 *E. coli* K12 genes (6.57% of all 4140 genes, 201 genes with functional annotations) and 392 (7.88% of 4971 genes, 273 with functional annotations) from further analyses. The majority of these genes encode hypothetical proteins, prophage proteins, ribosomal proteins and insertion sequences.

We used a sequence coverage-based strategy to exclude from further analysis (i) genes prone to alignment artefacts or (ii) subject to historical gene transfer events that occurred before our experiments with the K and W ancestral strains. To this end, we aligned reads of our K and W recipient ancestors to the *E. coli* K12, B and W reference genome (Table S1), and assigned the likely genomic origin of each gene in the ancestral recipient strain . Based on this analysis, we eliminated 27 and 78 genes in the ancestral K and W recipients, respectively, from further analysis, because they appeared to originate from strains other than the recipient. These genes were generally scattered across the genome, clustering physically only if they resided in the same operon.

**One-to-one orthologue identification**

To identify one-to-one orthologues, we first created pairwise genome alignments between the reference genomes of *E. coli* K12, B, and W using progressiveMauve [23] with default parameters, and then identified gene orthologues according to NCBI RefSeq annotations [24] based on synteny (Table S2). We eliminated genes that had tandem duplicates in one of the genomes (three out of 5669 genes). In addition to using annotated RefSeq features, we identified short repetitive regions using Tandem repeat finder [25].

**Identification of horizontally acquired genes using single nucleotide polymorphisms**

In a clone that resulted from a recombination event between a (non-identical) donor-recipient pair, any one gene can fall into three classes of sequences. First, its sequence could be the same as in the ancestral recipient but different from the ancestral donor, which makes an origin in the recipient likely (no horizontal transfer). Second, the gene may differ from both ancestors, which implies *de novo* mutations and a potential origin in either ancestor. Third, the gene could be the same as in the ancestral donor but different from the ancestral recipient, which makes an origin in the donor, and thus horizontal transfer likely. We focused on the last category, and required for a gene to be assigned an origin in the donor that at least three nucleotide sites within 1 kbp had the same genotype as the ancestral donor, but differed from the ancestral recipient.

More specifically, we screened for single-nucleotide variant sites where the ancestral recipient and donor had different genotypes, and used a protein coding gene’s codon position at the variant site inferred by snpEff [22] in one genome to identify the equivalent variant site in the other genome. Additionally, we only focused on those variant sites where the genotypes of the clone and both ancestors had been consistently called from the two alignments. Cases of inconsistently called genotypes were mainly due to deletion (> 50 bp) in either of the ancestors, leading to a variant called only in one alignment but not both. We constrained our analyses to synonymous or non-synonymous variant sites located within coding regions of genes, because we expected that the snpEff inference of mutation’s position would be most accurate within genes.

This approach allowed us to assign a donor origin to between 1594 and 2688 genes, for experiments where the donor and recipient were different (Table S6, lower part). Where the donor and recipient were identical, the approach proved unreliable, because it could identify very few genes as transferred. This is expected, because the two genomes are almost identical (Table S6, lower part). We did not analyze the corresponding sequence data further.

**Reference**

1. Datsenko KA, Wanner BL. One-step inactivation of chromosomal genes in Escherichia coli K-12 using PCR products. Proc. Natl. Acad. Sci. National Acad Sciences; 2000;97:6640–5.

2. Rao DN, Dryden DTF, Bheemanaik S. Type III restriction-modification enzymes: a historical perspective. Nucleic Acids Res. Oxford Univ Press; 2014;42:45–55.

3. Achtman M, Kennedy N, Skurray R. Cell--cell interactions in conjugating Escherichia coli: role of traT protein in surface exclusion. Proc. Natl. Acad. Sci. National Acad Sciences; 1977;74:5104–8.

4. Winkler J, Kao KC. Harnessing recombination to speed adaptive evolution in Escherichia coli. Metab. Eng. Elsevier; 2012;14:487–95.

5. Gellert M, Mizuuchi K, O’Dea MH, Itoh T, Tomizawa J-I. Nalidixic acid resistance: a second genetic character involved in DNA gyrase activity. Proc. Natl. Acad. Sci. National Acad Sciences; 1977;74:4772–6.

6. Jin DJ, Gross CA. Mapping and sequencing of mutations in the Escherichia coli rpoB gene that lead to rifampicin resistance. J. Mol. Biol. 1988;202:45–58.

7. Karczmarczyk M, Martins M, Quinn T, Leonard N, Fanning S. Mechanisms of fluoroquinolone resistance in Escherichia coli isolates from food-producing animals. Appl. Environ. Microbiol. Am Soc Microbiol; 2011;77:7113–20.

8. Campbell EA, Korzheva N, Mustaev A, Murakami K, Nair S, Goldfarb A, et al. Structural mechanism for rifampicin inhibition of bacterial RNA polymerase. Cell. Elsevier; 2001;104:901–12.

9. Phornphisutthimas S, Thamchaipenet A, Panijpan B. Conjugation in Escherichia coli. Biochem. Mol. Biol. Educ. Wiley Online Library; 2007;35:440–5.

10. Sprouffske K, Wagner A. Growthcurver: an R package for obtaining interpretable metrics from microbial growth curves. BMC Bioinformatics. 2016;17:172.

11. Baumler DJ, Peplinski RG, Reed JL, Glasner JD, Perna NT. The evolution of metabolic networks of E. coli. BMC Syst. Biol. BioMed Central; 2011;5:1.

12. Yoon SH, Han M-J, Jeong H, Lee CH, Xia X-X, Lee D-H, et al. Comparative multi-omics systems analysis of Escherichia coli strains B and K-12. Genome Biol. BioMed Central; 2012;13:1.

13. Monk JM, Charusanti P, Aziz RK, Lerman JA, Premyodhin N, Orth JD, et al. Genome-scale metabolic reconstructions of multiple Escherichia coli strains highlight strain-specific adaptations to nutritional environments. Proc. Natl. Acad. Sci. National Acad Sciences; 2013;110:20338–43.

14. R Core Team. R: A Language and Environment for Statistical Computing. Vienna, Austria; 2015; Available from: http://www.r-project.org/

15. Bauer AP, Dieckmann SM, Ludwig W, Schleifer K-H. Rapid identification of Escherichia coli safety and laboratory strain lineages based on Multiplex-PCR. FEMS Microbiol. Lett. The Oxford University Press; 2007;269:36–40.

16. Bolger AM, Lohse M, Usadel B. Trimmomatic: a flexible trimmer for {I}llumina sequence data. Bioinformatics. Oxford Univ Press; 2014;btu170.

17. Langmead B, Salzberg SL. Fast gapped-read alignment with {B}owtie 2. Nat Methods. Nature Publishing Group; 2012;9:357–9.

18. Riley M, Abe T, Arnaud MB, Berlyn MKB, Blattner FR, Chaudhuri RR, et al. Escherichia coli K-12: A cooperatively developed annotation snapshot - 2005. Nucleic Acids Res. 2006;34:1–9.

19. Archer CT, Kim JF, Jeong H, Park JH, Vickers CE, Lee SY, et al. The genome sequence of E. coli W (ATCC 9637): comparative genome analysis and an improved genome-scale reconstruction of E. coli. BMC Genomics. BioMed Central; 2011;12:1.

20. McKenna A, Hanna M, Banks E, Sivachenko A, Cibulskis K, Kernytsky A, et al. The Genome Analysis Toolkit: a MapReduce framework for analyzing next-generation DNA sequencing data. Genome Res. Cold Spring Harbor Lab; 2010;20:1297–303.

21. Auwera GA, Carneiro MO, Hartl C, Poplin R, del Angel G, Levy-Moonshine A, et al. From FastQ data to high-confidence variant calls: the genome analysis toolkit best practices pipeline. Curr. Protoc. Bioinforma. Wiley Online Library; 2013;10–1.

22. Cingolani P, Platts A, Wang LL, Coon M, Nguyen T, Wang L, et al. A program for annotating and predicting the effects of single nucleotide polymorphisms, SnpEff. Fly (Austin). [Internet]. Taylor & Francis; 2012;6:80–92. Available from: http://www.tandfonline.com/doi/abs/10.4161/fly.19695

23. Darling AE, Mau B, Perna NT. Progressive Mauve: Multiple alignment of genomes with gene flux and rearrangement. Genome [Internet]. 2009; Available from: http://arxiv.org/abs/0910.5780

24. Tatusova T, DiCuccio M, Badretdin A, Chetvernin V, Nawrocki EP, Zaslavsky L, et al. NCBI prokaryotic genome annotation pipeline. Nucleic Acids Res. Oxford Univ Press; 2016;gkw569.

25. Benson G. Tandem repeats finder: a program to analyze DNA sequences. Nucleic Acids Res. [Internet]. 1999;27:573–80. Available from: http://www.ncbi.nlm.nih.gov/pmc/articles/PMC148217/
