# Supplementary material for: Assessing the benefits of horizontal gene transfer by laboratory evolution and genome sequencing
Source: BMC Evol Biol. 2018 Apr 19;18:54. doi: 10.1186/s12862-018-1164-7 (PMC5909237; doi:10.1186/s12862-018-1164-7)
Supplement: Supplementary file 8 — Table S7. Statistical tests conducted. df: degrees of freedom, n = number of samples. * Statistical test adopted from [103], methods S1. The Mann-Whitney U-test is computed using the package exactRankTests [104] in R. '$\operatorname{Re}{\mathrm{c}}_{\mathrm{Y}}^{\mathrm{X}}$RecYX' denotes a population of Y recipient strains exposed to donor strain X. (DOCX 21 kb) [file 12862_2018_1164_MOESM8_ESM.docx]

| Test | Hypothesis | $\mathrm{Groups}$ | Statistical test | Parameters | p-value |
| --- | --- | --- | --- | --- | --- |
| 1 | Higher fractions of HPA adapted clones within population | $\mathrm{Re}c_{K} vs Rec_{K}^{K}$ | One-sided Mann-Whitney U-test | n=7 | 0.43 |
| 2 | Higher fractions of HPA adapted clones within population | $\mathrm{Re}c_{K}^{W} vs Rec_{K}^{K}$ | One-sided Mann-Whitney U-test | n=9 | 0.0079 |
| 3 | Higher fractions of HPA adapted clones within population | $\mathrm{Re}c_{K}^{B} vs Rec_{K}^{K}$ | One-sided Mann-Whitney U-test | n=10 | 0.0048 |
| 4 | Higher fractions of HPA adapted clones within population | $\mathrm{Re}c_{K}^{B} vs Rec_{K}^{W}$ | One-sided Mann-Whitney U-test | n=11 | 0.089 |
| 5 | Higher fitness values of K recipient populations recombined with different donors | $\mathrm{Re}c_{K}^{W} vs Rec_{K}^{K}$ | One-sided Mann-Whitney U-test | n=8 | 0.018 |
| 6 | Higher fitness values of K recipient populations recombined with different donors | $\mathrm{Re}c_{K}^{B} vs Rec_{K}^{K}$ | One-sided Mann-Whitney U-test | n=9 | 0.012 |
| 7 | Higher fitness values of K recipient populations recombined with different donors | $\mathrm{Re}c_{K}^{W} vs Rec_{K}$ | One-sided Mann-Whitney U-test | n=8 | 0.0018 |
| 8 | Higher fitness values of K recipient populations recombined with different donors | $\mathrm{Re}c_{K}^{B} vs Rec_{K}$ | One-sided Mann-Whitney U-test | n=9 | 0.012 |
| 9 | Different fitness values of K recipient populations recombined with different donors | $\mathrm{Re}c_{K}^{B} vs Rec_{K}^{W}$ | Two-sided Mann-Whitney U-test | n=11 | 0.16 |
| 10 | Higher fitness values of K recipient clones recombined with different donors | $\mathrm{Re}c_{K}^{K} vs Rec_{K}$ | One-sided Mann-Whitney U-test | n=28 | 0.92 |
| 11 | Different fitness values of K recipient clones recombined with different donors | $\mathrm{Re}c_{K}^{B} vs Rec_{K}^{W}$ | Two-sided Mann-Whitney U-test | n=11 | 0.41 |
| 12 | Different number of gene orthologues transferred from B donor to K recipient to that expecte by chance | $-$ | Pearson χ2 | df =1 | 0.67 |
| 13 | Different repetitive element occurrence in breakpoints regions than by chance | $\mathrm{Re}c_{K}^{B}$ breakpoint regions vs randomly drawn regions from the E. coli K 12 genome | Two-sided Mann-Whitney U-test | n=256 | 0.0026 |
| 14 | Higher repetitive element occurrence in breakpoints regions than by chance | $\mathrm{Re}c_{K}^{B}$ breakpoint regions vs randomly drawn regions from the E. coli K 12 genome | One-sided Mann-Whitney U-test | n=256 | 0.00044 |
| 15 | Lower SNP density between E. coli K12 and B genome in breakpoints regions than by chance | $\mathrm{Re}c_{K}^{B}$ breakpoint regions vs randomly drawn regions from the E. coli K 12 genome | One-sided Mann-Whitney U-test | n=256 | 0.45 |
| 16 | Different number of gene orthologues transferred from W donor to K recipient to that expected by chance | $-$ | Pearson χ2 | df =1 | 0.37 |
| 17 | Different repetitive element occurrence in breakpoints regions than by chance | $\mathrm{Re}c_{K}^{W}$ breakpoint regions vs randomly drawn regions from the E. coli K 12 genome | Mann-Whitney U-test | n=50 | 0.17 |
| 18 | Different SNP density between E. coli K12 and W genome in breakpoints regions than by chance | $\mathrm{Re}c_{K}^{W}$ breakpoint regions vs randomly drawn regions from the E. coli K 12 genome | Mann-Whitney U-test | n=50 | 0.11 |
| 19 | Higher number of 222 overlapping horizontal genes from the B and the W donor than by chance | $-$ | *Randomization test for overlap, with negative control iterated 1 x 105 times. | Number of W donor HGT genes = 321, Number of B donor HGT genes = 2649. | 7 x 10^-5^ |
| 20 | Higher fractions of butyric acid adapted clones within population | $\mathrm{Re}c_{W}^{W} vs Rec_{W}$ | One-sided Mann-Whitney U-test | n=6 | 0.65 |
| 21 | Higher fractions of butyric acid adapted clones within population | $\mathrm{Re}c_{W}^{B} vs Rec_{W}$ | One-sided Mann-Whitney U-test | n=8 | 0.32 |
| 22 | Higher fractions of butyric acid adapted clones within population | $\mathrm{Re}c_{W}^{K} vs Rec_{W}$ | One-sided Mann-Whitney U-test | n=8 | 0.50 |
| 23 | Higher fractions of butyric acid adapted clones within population | $\mathrm{Re}c_{W}^{B} vs Rec_{W}^{W}$ | One-sided Mann-Whitney U-test | n=8 | 0.32 |
| 24 | Higher fractions of butyric acid adapted clones within population | $\mathrm{Re}c_{W}^{K} vs Rec_{W}^{W}$ | One-sided Mann-Whitney U-test | n=8 | 0.45 |
| 25 | Higher fractions of butyric acid adapted clones within population | $\mathrm{Re}c_{W}^{B} vs Rec_{W}^{K}$ | One-sided Mann-Whitney U-test | n=10 | 0.45 |
| 26 | Higher fitness values of W recipient populations recombined with different donors | $\mathrm{Re}c_{W}^{W} vs Rec_{W}$ | One-sided Mann-Whitney U-test | n=6 | 0.60 |
| 27 | Higher fitness values of W recipient populations recombined with different donors | $\mathrm{Re}c_{W}^{B} vs Rec_{W}$ | One-sided Mann-Whitney U-test | n=11 | 0.50 |
| 28 | Higher fitness values of W recipient populations recombined with different donors | $\mathrm{Re}c_{W}^{K} vs Rec_{W}$ | One-sided Mann-Whitney U-test | n=8 | 0.79 |
| 29 | Higher fitness values of W recipient populations recombined with different donors | $\mathrm{Re}c_{W}^{B} vs Rec_{W}^{W}$ | One-sided Mann-Whitney U-test | n=8 | 0.61 |
| 30 | Higher fitness values of W recipient populations recombined with different donors | $\mathrm{Re}c_{W}^{K} vs Rec_{W}^{W}$ | One-sided Mann-Whitney U-test | n=8 | 0.79 |
| 31 | Higher fitness values of W recipient clones recombined with different donors | $\mathrm{Re}c_{W}^{B} vs Rec_{W}$ | One-sided Mann-Whitney U-test | n=29 | 0.00068 |
| 32 | Higher fitness values of W recipient clones recombined with different donors | $\mathrm{Re}c_{W}^{K} vs Rec_{W}$ | One-sided Mann-Whitney U-test | n=32 | 0.022 |
| 33 | Higher fitness values of W recipient clones recombined with different donors | $\mathrm{Re}c_{W}^{B} vs Rec_{W}^{W}$ | One-sided Mann-Whitney U-test | n=32 | 0.0018 |
| 34 | Higher fitness values of W recipient clones recombined with different donors | $\mathrm{Re}c_{W}^{K} vs Rec_{W}^{W}$ | One-sided Mann-Whitney U-test | n=32 | 0.084 |
| 35 | Higher fitness values of W recipient clones recombined with different donors | $\mathrm{Re}c_{W}^{B} vs Rec_{W}^{K}$ | Two-sided Mann-Whitney U-test | n=40 | 0.063 |
|  |  |  |  |  |  |
