# Supplementary material for: Assessing the benefits of horizontal gene transfer by laboratory evolution and genome sequencing
Source: BMC Evol Biol. 2018 Apr 19;18:54. doi: 10.1186/s12862-018-1164-7 (PMC5909237; doi:10.1186/s12862-018-1164-7)
Supplement: Supplementary file 9 — Table S12. Summary of growth parameters of HPA-evolved clones. We randomly selected four clones from each HPA-evolved population (Methods) and measured the clones’ growth in HPA-supplemented liquid media. The mean and standard deviations of growth rate, carrying capacity and area under the growth curve estimated by Growthcurver v0.2.1 are summarized for three replicate measurements and rounded to three significant digits. (DOCX 18 kb) [file 12862_2018_1164_MOESM9_ESM.docx]

| Population | Clone number | Mean growth rate | Mean carrying capacity | Mean area under the curve | Standard deviation of growth rate | Standard deviation of carrying capacity | Standard deviation of area under the growth curve |
| --- | --- | --- | --- | --- | --- | --- | --- |
| $\mathrm{Re}c_{K}^{W}$ 1 | 1 | 0.300 | 0.685 | 21.1 | 0.143 | 0.0464 | 0.741 |
| $\mathrm{Re}c_{K}^{W}$ 1 | 2 | 0.300 | 0.725 | 24.9 | 0.0485 | 0.179 | 5.35 |
| $\mathrm{Re}c_{K}^{W}$ 1 | 3 | 0.355 | 0.725 | 25.4 | 0.112 | 0.139 | 3.85 |
| $\mathrm{Re}c_{K}^{W}$ 1 | 4 | 0.368 | 0.668 | 22.4 | 0.134 | 0.226 | 6.56 |
| $\mathrm{Re}c_{K}^{B}$ 1 | 1 | 0.313 | 0.865 | 29.4 | 0.0369 | 0.032 | 1.83 |
| $\mathrm{Re}c_{K}^{B}$ 1 | 2 | 0.276 | 0.902 | 29.2 | 0.0307 | 0.0243 | 2.00 |
| $\mathrm{Re}c_{K}^{B}$ 1 | 3 | 0.474 | 0.761 | 26.9 | 0.155 | 0.128 | 3.14 |
| $\mathrm{Re}c_{K}^{B}$ 1 | 4 | 0.471 | 0.765 | 25.8 | 0.261 | 0.171 | 4.65 |
| $\mathrm{Re}c_{K}^{B}$ 2 | 1 | 0.293 | 0.684 | 23.7 | 0.0764 | 0.13 | 5.03 |
| $\mathrm{Re}c_{K}^{B}$ 2 | 2 | 0.349 | 0.779 | 26.1 | 0.139 | 0.107 | 2.09 |
| $\mathrm{Re}c_{K}^{B}$ 2 | 3 | 0.38 | 0.69 | 24.3 | 0.119 | 0.103 | 3.67 |
| $\mathrm{Re}c_{K}^{B}$ 2 | 4 | 0.473 | 0.644 | 22.6 | 0.234 | 0.149 | 3.00 |
| $\mathrm{Re}c_{K}^{B}$ 3 | 1 | 0.294 | 0.931 | 30.2 | 0.0285 | 0.0427 | 0.295 |
| $\mathrm{Re}c_{K}^{B}$ 3 | 2 | 0.313 | 0.896 | 29.0 | 0.0367 | 0.0654 | 2.77 |
| $\mathrm{Re}c_{K}^{B}$ 3 | 3 | 0.328 | 0.869 | 28.2 | 0.0574 | 0.0382 | 1.81 |
| $\mathrm{Re}c_{K}^{B}$ 3 | 4 | 0.398 | 0.721 | 24.2 | 0.147 | 0.0878 | 1.98 |
| $\mathrm{Re}c_{K}^{K}$ 1 | 1 | 0.153 | 0.0168 | 0.746 | 0.265 | 0.0067 | 0.314 |
| $\mathrm{Re}c_{K}^{K}$ 1 | 2 | 0.0911 | 0.0207 | 0.888 | 0.158 | 0.0146 | 0.581 |
| $\mathrm{Re}c_{K}^{K}$ 1 | 3 | 0.0882 | 0.0137 | 0.599 | 0.153 | 0.00326 | 0.143 |
| $\mathrm{Re}c_{K}^{K}$ 1 | 4 | 0.0394 | 0.0203 | 0.958 | 0.0682 | 0.0169 | 0.82 |
| $\mathrm{Re}c_{K}^{K}$ 2 | 1 | 0 | 0.0127 | 0.576 | 0 | 0.00725 | 0.351 |
| $\mathrm{Re}c_{K}^{K}$ 2 | 2 | 0 | 0.0118 | 0.542 | 0 | 0.00573 | 0.275 |
| $\mathrm{Re}c_{K}^{K}$ 2 | 3 | 0.300 | 0.501 | 13.0 | 0.272 | 0.432 | 11.6 |
| $\mathrm{Re}c_{K}^{K}$ 2 | 4 | 0 | 0.00639 | 0.296 | 0 | 0.00625 | 0.285 |
| $\mathrm{Re}c_{K}^{K}$ 3 | 1 | 0.105 | 0.0117 | 0.496 | 0.182 | 0.00586 | 0.226 |
| $\mathrm{Re}c_{K}^{K}$ 3 | 2 | 0 | 0.0094 | 0.418 | 0 | 0.00494 | 0.180 |
| $\mathrm{Re}c_{K}^{K}$ 3 | 3 | 0.180 | 0.204 | 4.34 | 0.312 | 0.335 | 6.63 |
| $\mathrm{Re}c_{K}^{K}$ 3 | 4 | 0.0642 | 0.002 | 0.341 | 0.111 | 0.200 | 0.225 |
| $\mathrm{Re}c_{K}$ 3 | 1 | 0.0475 | 0.00457 | 0.213 | 0.0823 | 0.00471 | 0.209 |
| $\mathrm{Re}c_{K}$ 3 | 2 | 0.196 | 0.0101 | 0.435 | 0.241 | 0.00255 | 0.115 |
| $\mathrm{Re}c_{K}$ 3 | 3 | 0 | 0.00379 | 0.172 | 0 | 0.00656 | 0.299 |
| $\mathrm{Re}c_{K}$ 3 | 4 | 0.179 | 14000 | 0.499 | 0.264 | 24200 | 0.0498 |
| $\mathrm{Re}c_{K}^{W}$ 4 | 1 | 0.562 | 0.656 | 24.1 | 0.2 | 0.143 | 4.70 |
| $\mathrm{Re}c_{K}^{W}$ 4 | 2 | 0.682 | 0.609 | 22.5 | 0.216 | 0.0871 | 3.21 |
| $\mathrm{Re}c_{K}^{W}$ 4 | 3 | 0.674 | 0.600 | 22.2 | 0.195 | 0.0986 | 3.56 |
| $\mathrm{Re}c_{K}^{W}$ 4 | 4 | 0.617 | 0.531 | 19.2 | 0.150 | 0.0747 | 3.09 |
| $\mathrm{Re}c_{K}^{W}$ 5 | 1 | 0.0763 | 0.287 | 6.58 | 0.132 | 0.467 | 10.1 |
| $\mathrm{Re}c_{K}^{W}$ 5 | 2 | 0.388 | 0.688 | 20.4 | 0.113 | 0.171 | 5.87 |
| $\mathrm{Re}c_{K}^{W}$ 5 | 3 | 0.145 | 190 | 0.582 | 0.248 | 329 | 0.213 |
| $\mathrm{Re}c_{K}^{W}$ 5 | 4 | 0.146 | 2410 | 0.391 | 0.228 | 4180 | 0.235 |
| $\mathrm{Re}c_{K}^{W}$ 6 | 1 | 0.463 | 0.599 | 20.4 | 0.202 | 0.251 | 7.71 |
| $\mathrm{Re}c_{K}^{W}$ 6 | 2 | 0.319 | 0.646 | 21.2 | 0.0816 | 0.164 | 5.67 |
| $\mathrm{Re}c_{K}^{W}$ 6 | 3 | 0.327 | 0.593 | 19.9 | 0.133 | 0.095 | 3.10 |
| $\mathrm{Re}c_{K}^{W}$ 6 | 4 | 0.304 | 0.673 | 21.7 | 0.0934 | 0.195 | 4.89 |
| $\mathrm{Re}c_{K}^{B}$ 4 | 1 | 0.489 | 0.641 | 20.2 | 0.142 | 0.137 | 3.30 |
| $\mathrm{Re}c_{K}^{B}$ 4 | 2 | 0.362 | 0.774 | 24.9 | 0.0679 | 0.0478 | 1.28 |
| $\mathrm{Re}c_{K}^{B}$ 4 | 3 | 0.382 | 0.726 | 23.4 | 0.064 | 0.0909 | 2.23 |
| $\mathrm{Re}c_{K}^{B}$ 4 | 4 | 0.435 | 0.773 | 25.1 | 0.146 | 0.117 | 2.02 |
| $\mathrm{Re}c_{K}^{B}$ 5 | 1 | 0.294 | 0.626 | 18.0 | 0.0268 | 0.0723 | 2.18 |
| $\mathrm{Re}c_{K}^{B}$ 5 | 2 | 0.283 | 0.754 | 22.1 | 0.0744 | 0.111 | 3.18 |
| $\mathrm{Re}c_{K}^{B}$ 5 | 3 | 0.278 | 0.772 | 24.0 | 0.0505 | 0.0458 | 0.474 |
| $\mathrm{Re}c_{K}^{B}$ 5 | 4 | 0.266 | 0.766 | 22.4 | 0.0739 | 0.0695 | 2.72 |
| $\mathrm{Re}c_{K}^{B}$ 6 | 1 | 0.37 | 0.69 | 23.2 | 0.105 | 0.274 | 8.05 |
| $\mathrm{Re}c_{K}^{B}$ 6 | 2 | 0.289 | 0.916 | 29.5 | 0.0307 | 0.0736 | 1.38 |
| $\mathrm{Re}c_{K}^{B}$ 6 | 3 | 0.42 | 0.813 | 26.4 | 0.138 | 0.188 | 5.00 |
| $\mathrm{Re}c_{K}^{B}$ 6 | 4 | 0.466 | 0.518 | 17.4 | 0.163 | 0.107 | 2.98 |
| $\mathrm{Re}c_{K}^{K}$ 5 | 1 | 0.238 | 0.0133 | 0.573 | 0.211 | 0.00796 | 0.335 |
| $\mathrm{Re}c_{K}^{K}$ 5 | 2 | 0.0918 | 0.200 | 0.474 | 0.158 | 0.410 | 0.209 |
| $\mathrm{Re}c_{K}^{K}$ 5 | 3 | 0 | 0.00886 | 0.38 | 0 | 0.00793 | 0.337 |
| $\mathrm{Re}c_{K}^{K}$ 5 | 4 | 0.112 | 38.1 | 0.388 | 0.192 | 66.0 | 0.153 |
| $\mathrm{Re}c_{K}$ 5 | 1 | 0.382 | 0.774 | 21.2 | 0.167 | 0.117 | 4.96 |
| $\mathrm{Re}c_{K}$ 5 | 2 | 0.168 | 0.0682 | 0.975 | 0.146 | 0.094 | 0.696 |
| $\mathrm{Re}c_{K}$ 5 | 3 | 0.385 | 0.374 | 8.66 | 0.167 | 0.318 | 7.26 |
| $\mathrm{Re}c_{K}$ 5 | 4 | 0.286 | 0.622 | 14.8 | 0.058 | 0.191 | 6.96 |
| $\mathrm{Re}c_{K}$ 6 | 1 | 0.00309 | 311 | 0.371 | 0.00535 | 538 | 0.145 |
| $\mathrm{Re}c_{K}$ 6 | 2 | 0.142 | 0.0117 | 0.531 | 0.247 | 0.00422 | 0.188 |
| $\mathrm{Re}c_{K}$ 6 | 3 | 0 | 0.0134 | 0.61 | 0 | 0.0024 | 0.0834 |
| $\mathrm{Re}c_{K}$ 6 | 4 | 0.137 | 0.00987 | 0.441 | 0.237 | 0.00861 | 0.383 |
