# Supplementary material for: Assessing the benefits of horizontal gene transfer by laboratory evolution and genome sequencing
Source: BMC Evol Biol. 2018 Apr 19;18:54. doi: 10.1186/s12862-018-1164-7 (PMC5909237; doi:10.1186/s12862-018-1164-7)
Supplement: Supplementary file 12 — Table S16. Summary of data from horizontally transferred genes for each analysed clone in populations where horizontal gene transfer was detected. The table shows the number of unique genes and one-to-one orthologues introduced from the donor, the number of single nucleotide polymorphisms introduced by horizontal gene transfer from the donor genome, the number of de novo mutations at recipient background. (DOCX 15 kb) [file 12862_2018_1164_MOESM12_ESM.docx]

| clone | Experiment | Number of unique genes introduced from donor | Number of transferred genes with a one-to-one orthologue in the recipient genome | *De-novo* mutations identified in recipient background |
| --- | --- | --- | --- | --- |
| S01_4H1_A1_A1 | $\mathrm{Re}c_{K}^{W}$ | 26 | 35 | 1 |
| S02_4H1_C1_A4 | $\mathrm{Re}c_{K}^{B}$ | 15 | 34 | 1 |
| S03_4H1_C3_A5 | $\mathrm{Re}c_{K}^{B}$ | 163 | 1297 | 6 |
| S06_4H1_A3_B2 | $\mathrm{Re}c_{K}^{W}$ | 27 | 35 | 2 |
| S07_4H1_C1_B4 | $\mathrm{Re}c_{K}^{B}$ | 16 | 40 | 13 |
| S08_4H1_C3_B5 | $\mathrm{Re}c_{K}^{B}$ | 163 | 1288 | 5 |
| S09_4H1_C5_B6 | $\mathrm{Re}c_{K}^{B}$ | 87 | 713 | 15 |
| S13_4H1_A5_C3 | $\mathrm{Re}c_{K}^{W}$ | 33 | 130 | 0 |
| S14_4H1_C5_C6 | $\mathrm{Re}c_{K}^{B}$ | 87 | 696 | 17 |
| S16_4H1_A1_D1 | $\mathrm{Re}c_{K}^{W}$ | 27 | 34 | 2 |
| S17_4H1_A3_D2 | $\mathrm{Re}c_{K}^{W}$ | 25 | 12 | 2 |
| S24_4H2_A5_E3 | $\mathrm{Re}c_{K}^{W}$ | 33 | 120 | 0 |
| S25_4H2_C1_E4 | $\mathrm{Re}c_{K}^{B}$ | 112 | 1186 | 13 |
| S30_4H2_A1_F1 | $\mathrm{Re}c_{K}^{W}$ | 52 | 235 | 0 |
| S31_4H2_A5_F3 | $\mathrm{Re}c_{K}^{W}$ | 36 | 121 | 0 |
| S32_4H2_C1_F4 | $\mathrm{Re}c_{K}^{B}$ | 114 | 1188 | 8 |
| S33_4H2_C3_F5 | $\mathrm{Re}c_{K}^{B}$ | 203 | 1956 | 4 |
| S34_4H2_C5_F6 | $\mathrm{Re}c_{K}^{B}$ | 144 | 1237 | 5 |
| S37_4H2_A1_G1 | $\mathrm{Re}c_{K}^{W}$ | 50 | 230 | 1 |
| S39_4H2_C3_G5 | $\mathrm{Re}c_{K}^{B}$ | 200 | 1941 | 3 |
| S40_4H2_C5_G6 | $\mathrm{Re}c_{K}^{B}$ | 109 | 1176 | 4 |
| S48_BU1_C3_B5 | $\mathrm{Re}c_{W}^{K}$ | 0 | 0 | 4 |
| S53_BU1_C3_C5 | $\mathrm{Re}c_{W}^{K}$ | 0 | 0 | 4 |
| S54_BU1_C5_C6 | $\mathrm{Re}c_{W}^{K}$ | 0 | 0 | 2 |
| S59_BU1_C5_D6 | $\mathrm{Re}c_{W}^{K}$ | 0 | 0 | 2 |
| S64_BU2_C1_E4 | $\mathrm{Re}c_{W}^{K}$ | 33 | 183 | 17 |
| S69_BU2_C1_F4 | $\mathrm{Re}c_{W}^{K}$ | 39 | 184 | 7 |
| S69_BU2_C1_F4 | $\mathrm{Re}c_{W}^{K}$ | 0 | 0 | 15 |
| S70_BU2_C3_F5 | $\mathrm{Re}c_{W}^{K}$ | 0 | 0 | 7 |
| S71_BU2_C5_F6 | $\mathrm{Re}c_{W}^{K}$ | 0 | 0 | 6 |
| S78_BU2_C3_G5 | $\mathrm{Re}c_{W}^{K}$ | 0 | 0 | 23 |
