# Supplementary material for: Assessing the benefits of horizontal gene transfer by laboratory evolution and genome sequencing
Source: BMC Evol Biol. 2018 Apr 19;18:54. doi: 10.1186/s12862-018-1164-7 (PMC5909237; doi:10.1186/s12862-018-1164-7)
Supplement: Supplementary file 16 — Text S2. Genes transferred to the K recipient from only one but not both donors during evolution on HPA (DOCX 12 kb) [file 12862_2018_1164_MOESM16_ESM.docx]

## S2 Text. Genes transferred to the K recipient from only one but not both donors during evolution on HPA

In our HPA experiment, we studied genes horizontally transferred specifically into the K recipient strain from the B or the W donor (bot not from both). Specifically, we identified 269 genes transferred from the E. *coli* B donor to at least one K recipient clone, which were present in the *E. coli* B but not in the K12 genome. Most of these genes (213) encode either hypothetical or bacteriophage proteins, and come from four regions in the *E. coli* B genome (700 - 900kbp, 2.11 - 2.10 - 2.11 Mb, 2.82 - 3.06 Mb, 3.77 - 3.99 Mb). Conversely, we observed 37 genes that were transferred from the *E. coli* W donor to at least one K recipient clone, which were present in the *E. coli* W but not in the K12 genome. Among these 37 genes, 17 have no functional annotation, another 17 genes fall into the *ets* operon (most of which encodes type six secretion proteins), two genes encode hypothetical proteins, one gene encodes the penicillin G cyclase, and one gene encodes an inner membrane protein [1]. None of these genes are known to contribute to HPA metabolism.

1. Tatusova T, DiCuccio M, Badretdin A, Chetvernin V, Nawrocki EP, Zaslavsky L, et al. NCBI prokaryotic genome annotation pipeline. Nucleic Acids Res. Oxford Univ Press; 2016;44:6614–24.
