# Supplementary material for: Assessing the benefits of horizontal gene transfer by laboratory evolution and genome sequencing
Source: BMC Evol Biol. 2018 Apr 19;18:54. doi: 10.1186/s12862-018-1164-7 (PMC5909237; doi:10.1186/s12862-018-1164-7)
Supplement: Supplementary file 18 — Table S8. Number of clones with a mutant allele in specific genes found in the HPA adapted populations. The coordinates of the start and the end of a gene are listed according to E. coli K12 reference genome coordinates. α: Number of variant sites identified within a gene among all HPA evolved clones. Β: Number of clones in a given recombination condition ('$\operatorname{Re}{\mathrm{c}}_{\mathrm{Y}}^{\mathrm{X}}$RecYX' , where X is the donor identity, and Y the recipient identity) with the derived allele(s) of the gene. (DOCX 15 kb) [file 12862_2018_1164_MOESM18_ESM.docx]

| Start | End | *Gene* | Protein | ^α^Number of variant sites | ^β^$\mathrm{Re}c_{K}^{W}$ | ^β^ $\mathrm{Re}c_{K}^{B}$ | ^β^ $\mathrm{Re}c_{K}^{K}$ | ^β^$\mathrm{Re}c_{K}$ |
| --- | --- | --- | --- | --- | --- | --- | --- | --- |
| 68348 | 70048 | *araB* | L-ribulokinase | 1 | 0 | 0 | 0 | 1 |
| 87357 | 87848 | *ilvH* | acetolactate synthase 3, small subunit, valine-sensitive | 1 | 2 | 0 | 0 | 0 |
| 435137 | 435556 | *nusB* | transcription antitermination protein | 1 | 2 | 0 | 0 | 0 |
| 721730 | 724414 | *kdpD* | fused sensory histidine kinase in two-component regulatory system with KdpE: signal sensing protein | 1 | 1 | 0 | 0 | 0 |
| 1102152 | 1102541 | *csgE* | curlin secretion specificity factor | 1 | 0 | 0 | 0 | 2 |
| 1141182 | 1144367 | *rne* | fused ribonucleaseE: endoribonuclease/RNA-binding protein/RNA degradosome binding protein | 4 | 0 | 2 | 2 | 1 |
| 1151939 | 1153180 | *fabF* | 3-oxoacyl-[acyl-carrier-protein] synthase II | 1 | 0 | 2 | 0 | 0 |
| 1362743 | 1364230 | *puuC* | gamma-glutamyl-gamma-aminobutyraldehyde dehydrogenase; succinate semialdehyde dehydrogenase | 1 | 0 | 0 | 0 | 2 |
| 1870385 | 1871860 | *yeaI* | putative membrane-anchored diguanylate cyclase | 2 | 0 | 1 | 0 | 0 |
| 2077569 | 2077943 | *cbtA* | CP4-44 prophage; toxin of the YeeV-YeeU toxin-antitoxin system | 1 | 0 | 1 | 0 | 0 |
| 2336793 | 2339420 | *gyrA* | DNA gyrase (type II topoisomerase), subunit A | 1 | 0 | 1 | 0 | 0 |
| 2966188 | 2968434 | *ptsP* | fused PTS enzyme: PEP-protein phosphotransferase (enzyme I)/GAF domain containing protein | 7 | 0 | 2 | 2 | 1 |
| 3166111 | 3167718 | *ygiS* | putative inner membrane ABC superfamily transporter permease | 1 | 0 | 0 | 2 | 0 |
| 3209530 | 3210543 | *tsaD* | tRNA(ANN) t(6)A37 threonylcarbamoyladenosine modification protein; glycation binding protein | 1 | 0 | 0 | 1 | 0 |
| 3316039 | 3317526 | *nusA* | transcription termination/antitermination L factor | 2 | 0 | 0 | 1 | 0 |
| 3347966 | 3348238 | *npr* | phosphohistidinoprotein-hexose phosphotransferase component of N-regulated PTS system (Npr) | 1 | 0 | 0 | 0 | 1 |
| 3676290 | 3678350 | *yhjG* | putative inner membrane-anchored periplasmic AsmA family protein | 1 | 0 | 0 | 0 | 1 |
| 4033145 | 4034596 | *trkH* | potassium transporter | 1 | 0 | 0 | 2 | 0 |
| 4180560 | 4180925 | *rplL* | 50S ribosomal subunit protein L7/L12 | 1 | 0 | 0 | 0 | 1 |
| 4181245 | 4185273 | *rpoB* | RNA polymerase, beta subunit | 3 | 0 | 0 | 2 | 1 |
| 4185350 | 4189573 | *rpoC* | RNA polymerase, beta prime subunit | 2 | 0 | 0 | 0 | 2 |
