# Supplementary material for: Assessing the benefits of horizontal gene transfer by laboratory evolution and genome sequencing
Source: BMC Evol Biol. 2018 Apr 19;18:54. doi: 10.1186/s12862-018-1164-7 (PMC5909237; doi:10.1186/s12862-018-1164-7)
Supplement: Supplementary file 20 — Table S13. Summary of growth parameters of populations evolved in butyric acid. The table shows mean and standard deviations of growth rate, carrying capacity and area under the growth curve estimated by Growthcurver v0.2.1 at the end of the butyric acid-adaptation experiment based on three replicate measurements of growth in butyric acid-supplemented liquid medium and rounded to three significant digits. (DOCX 13 kb) [file 12862_2018_1164_MOESM20_ESM.docx]

| Population | Mean growth rate | Mean carrying capacity | Mean area under the curve | Standard deviation of growth rate | Standard deviation of carrying capacity | Standard deviation of area under the growth curve |
| --- | --- | --- | --- | --- | --- | --- |
| $\mathrm{Re}c_{W}^{B}$ 2 | 0.0786 | 0.169 | 2.66 | 0.00411 | 0.0346 | 0.23 |
| $\mathrm{Re}c_{W}^{B}$ 3 | 0 | 0.134 | 5.69 | 0 | 0.00343 | 0.131 |
| $\mathrm{Re}c_{W}^{K}$ 2 | 0 | 0.0117 | 0.533 | 0 | 0.00195 | 0.0925 |
| $\mathrm{Re}c_{W}^{K}$ 3 | 0 | 0.0143 | 0.647 | 0 | 0.0011 | 0.0554 |
| $\mathrm{Re}c_{W}$ 2 | 0.0726 | 0.0189 | 0.838 | 0.126 | 0.00193 | 0.0901 |
| $\mathrm{Re}c_{W}^{B}$ 4 | 0.0229 | 0.0242 | 1.05 | 0.0397 | 0.00359 | 0.171 |
| $\mathrm{Re}c_{W}^{B}$ 5 | 0.258 | 0.0935 | 3.08 | 0.00357 | 0.00371 | 0.259 |
| $\mathrm{Re}c_{W}^{B}$ 6 | 0 | 0.0160 | 0.730 | 0 | 0.0013 | 0.0547 |
| $\mathrm{Re}c_{W}^{K}$ 4 | 0 | 0.0240 | 1.11 | 0 | 0.00295 | 0.148 |
| $\mathrm{Re}c_{W}^{K}$ 5 | 0.0424 | < 0.001 | 1.78 | 0.00467 | 0.010 | 0.323 |
| $\mathrm{Re}c_{W}^{K}$ 6 | 0.243 | 0.104 | 3.75 | 0.0209 | 0.00392 | 0.232 |
| $\mathrm{Re}c_{W}^{W}$ 4 | 0 | 0.00847 | 0.386 | 0 | 0.0147 | 0.669 |
| $\mathrm{Re}c_{W}^{W}$ 5 | 0.0477 | < 0.001 | 1.86 | 0.000718 | 0.200 | 0.389 |
| $\mathrm{Re}c_{W}^{W}$ 6 | 0.176 | 0.105 | 2.74 | 0.0280 | 0.0159 | 0.335 |
| $\mathrm{Re}c_{W}$ 4 | 0 | 0.0222 | 1.02 | 0 | 0.00264 | 0.113 |
| $\mathrm{Re}c_{W}$ 5 | 0.0649 | 0.224 | 2.33 | 0.00747 | 0.0681 | 0.305 |
