# Supplementary material for: Assessing the benefits of horizontal gene transfer by laboratory evolution and genome sequencing
Source: BMC Evol Biol. 2018 Apr 19;18:54. doi: 10.1186/s12862-018-1164-7 (PMC5909237; doi:10.1186/s12862-018-1164-7)
Supplement: Supplementary file 21 — Table S14. Summary of growth parameters of butyric acid-evolved clones. We randomly selected four clones from each butyric acid-evolved population (Methods) and measured the clones’ growth in butyric acid-supplemented liquid media. The mean and standard deviations of growth rate, carrying capacity and area under the growth curve estimated by Growthcurver v0.2.1 are summarized for three replicate measurements and rounded to three significant figures. (DOCX 17 kb) [file 12862_2018_1164_MOESM21_ESM.docx]

| Population | Clone number | Mean growth rate | Mean carrying capacity | Mean area under the growth curve | Standard deviation of growth rate | Standard deviation of carrying capacity | Standard deviation of area under the growth curve |
| --- | --- | --- | --- | --- | --- | --- | --- |
| $\mathrm{Re}c_{W}^{B}$ 2 | 1 | 0.120 | 0.13 | 2.58 | 0.0280 | 0.0553 | 0.183 |
| $\mathrm{Re}c_{W}^{B}$2 | 2 | 0.170 | 0.15 | 4.32 | 0.136 | 0.00579 | 1.33 |
| $\mathrm{Re}c_{W}^{B}$ 2 | 3 | 0.178 | 0.13 | 4.04 | 0.0900 | 0.0213 | 0.0350 |
| $\mathrm{Re}c_{W}^{B}$ 2 | 4 | 0.0380 | 0.138 | 4.46 | 0.0530 | 0.0261 | 2.74 |
| $\mathrm{Re}c_{W}^{B}$ 3 | 1 | 0.196 | 0.0719 | 2.88 | 0.278 | 0.00489 | 0.196 |
| $\mathrm{Re}c_{W}^{B}$ 3 | 2 | 0.213 | 0.0814 | 3.58 | 0.301 | 0.00328 | 0.7700 |
| $\mathrm{Re}c_{W}^{B}$ 3 | 3 | 0.220 | 0.0952 | 3.99 | 0.312 | 0.0241 | 1.33 |
| $\mathrm{Re}c_{W}^{B}$ 3 | 4 | 0.188 | 0.0786 | 3.23 | 0.266 | 0.00835 | 0.685 |
| $\mathrm{Re}c_{W}^{K}$ 2 | 1 | 0.051 | 0.0289 | 1.100 | 0.0200 | 0.0143 | 0.0150 |
| $\mathrm{Re}c_{W}^{K}$ 2 | 2 | 0.0410 | 0.0241 | 0.920 | 0.0530 | 0.034 | 0.0360 |
| $\mathrm{Re}c_{W}^{K}$2 | 3 | 0.0220 | 0.0566 | 0.592 | 0.0320 | 0.08 | 0.837 |
| $\mathrm{Re}c_{W}^{K}$ 2 | 4 | 0.0250 | 0.0368 | 0.684 | 0.0350 | 0.0521 | 0.967 |
| $\mathrm{Re}c_{W}^{K}$ 3 | 1 | 0.0720 | 0.0281 | 0.976 | 0.0140 | 0.0124 | 0.408 |
| $\mathrm{Re}c_{W}^{K}$ 3 | 2 | 0.0200 | 0.0373 | 0.649 | 0.0290 | 0.0528 | 0.918 |
| $\mathrm{Re}c_{W}^{K}$ 3 | 3 | 0.0350 | 0.0251 | 1.05 | 0.00700 | 0.0355 | 0.231 |
| $\mathrm{Re}c_{W}^{K}$ 3 | 4 | 0.0100 | 0.00995 | 1.01 | 0.0140 | 0.0141 | 0.153 |
| $\mathrm{Re}c_{W}$ 2 | 1 | 0.092 | 0.023 | 1.21 | 0.0890 | 0.0132 | 0.0940 |
| $\mathrm{Re}c_{W}$ 2 | 2 | 0.00600 | 0.0082 | 0.913 | 0.00800 | 0.0116 | 1.29 |
| $\mathrm{Re}c_{W}$ 2 | 3 | 0.0100 | 0.0092 | 1.01 | 0.0140 | 0.013 | 1.42 |
| $\mathrm{Re}c_{W}$ 2 | 4 | 0 | 0 | 0 | 0 | 0 | 0 |
| $\mathrm{Re}c_{W}^{B}$ 4 | 1 | 0.0670 | 0.158 | 3.22 | 0.0130 | 0.223 | 0.680 |
| $\mathrm{Re}c_{W}^{B}$ 4 | 2 | 0.132 | 0.155 | 3.14 | 0.0610 | 0.0851 | 0.757 |
| $\mathrm{Re}c_{W}^{B}$ 4 | 3 | 0.0800 | 0.0964 | 2.04 | 0.0530 | 0.123 | 1.83 |
| $\mathrm{Re}c_{W}^{B}$ 4 | 4 | 0.120 | 0.149 | 2.44 | 0.0770 | 0.064 | 0.392 |
| $\mathrm{Re}c_{W}^{B}$ 5 | 1 | 0.272 | 0.111 | 4.23 | 0.198 | 0.0346 | 2.00 |
| $\mathrm{Re}c_{W}^{B}$ 5 | 2 | 0.141 | 0.0177 | 0.636 | 0.199 | 0.0251 | 0.899 |
| $\mathrm{Re}c_{W}^{B}$ 5 | 3 | 0.078 | 0.0653 | 1.41 | 0.110 | 0.0924 | 2.00 |
| $\mathrm{Re}c_{W}^{B}$ 5 | 4 | 0.135 | 0.442 | 2.300 | 0.118 | 0.469 | 0.643 |
| $\mathrm{Re}c_{W}^{B}$ 6 | 1 | 0.00100 | 0 | 0.703 | 0.00100 | 0 | 0.995 |
| $\mathrm{Re}c_{W}^{B}$ 6 | 2 | 0.0160 | 0.0131 | 1.53 | 0.0220 | 0.0185 | 0.612 |
| $\mathrm{Re}c_{W}^{B}$ 6 | 3 | 0.0200 | 0.00948 | 1.15 | 0.0280 | 0.0134 | 0.401 |
| $\mathrm{Re}c_{W}^{B}$ 6 | 4 | 0.146 | 0.0557 | 2.33 | 0.207 | 0.0362 | 1.76 |
| $\mathrm{Re}c_{W}^{K}$ 4 | 1 | 0.0970 | 0.0688 | 2.54 | 0.118 | 0.0973 | 2.15 |
| $\mathrm{Re}c_{W}^{K}$ 4 | 2 | 0.113 | 0.0929 | 3.3 | 0.16 | 0.0213 | 0.084 |
| $\mathrm{Re}c_{W}^{K}$ 4 | 3 | 0.114 | 0.0516 | 1.55 | 0.162 | 0.0729 | 2.19 |
| $\mathrm{Re}c_{W}^{K}$ 4 | 4 | 0.072 | 0.0548 | 2.19 | 0.085 | 0.0775 | 0.735 |
| $\mathrm{Re}c_{W}^{K}$ 5 | 1 | 0.175 | 0.133 | 3.44 | 0.03 | 0.0207 | 1.49 |
| $\mathrm{Re}c_{W}^{K}$ 5 | 2 | 0.195 | 0.11 | 1.88 | 0.171 | 0.022 | 1.26 |
| $\mathrm{Re}c_{W}^{K}$ 5 | 3 | 0.033 | 0 | 1.25 | 0.025 | 0 | 0.039 |
| $\mathrm{Re}c_{W}^{K}$ 5 | 4 | 0.057 | 0.107 | 3.72 | 0.081 | 0.00279 | 1.45 |
| $\mathrm{Re}c_{W}^{K}$ 6 | 1 | 0.218 | 0.118 | 1.86 | 0.026 | 0.0327 | 0.1 |
| $\mathrm{Re}c_{W}^{K}$ 6 | 2 | 0.183 | 0.0492 | 1.92 | 0.258 | 0.0695 | 2.72 |
| $\mathrm{Re}c_{W}^{K}$ 6 | 3 | 0.059 | 0.139 | 3.02 | 0.008 | 0.197 | 2.05 |
| $\mathrm{Re}c_{W}^{K}$ 6 | 4 | 0.071 | 0.0424 | 1.27 | 0.026 | 0.0124 | 0.061 |
| $\mathrm{Re}c_{W}^{W}$ 4 | 1 | 0.047 | 0.0721 | 1.8 | 0.039 | 0.102 | 0.782 |
| $\mathrm{Re}c_{W}^{W}$ 4 | 2 | 0.012 | 0 | 1.66 | 0.007 | 0 | 0.723 |
| $\mathrm{Re}c_{W}^{W}$ 4 | 3 | 0.04 | 0.196 | 2.33 | 0.038 | 0.277 | 1.27 |
| $\mathrm{Re}c_{W}^{W}$ 4 | 4 | 0.023 | 0.019 | 0.553 | 0.033 | 0.0269 | 0.782 |
| $\mathrm{Re}c_{W}^{W}$ 5 | 1 | 0.014 | 0.179 | 4.58 | 0.02 | 0.0808 | 2.02 |
| $\mathrm{Re}c_{W}^{W}$ 5 | 2 | 0.129 | 0.139 | 2.96 | 0.04 | 0.0403 | 1.48 |
| $\mathrm{Re}c_{W}^{W}$ 5 | 3 | 0.101 | 0.0514 | 2.07 | 0.136 | 0.0727 | 0.778 |
| $\mathrm{Re}c_{W}^{W}$ 5 | 4 | 0.025 | 0.0013 | 0.521 | 0.036 | 0.00184 | 0.55 |
| $\mathrm{Re}c_{W}^{W}$ 6 | 1 | 0.062 | 0.0304 | 1.33 | 0.059 | 0.0429 | 0.094 |
| $\mathrm{Re}c_{W}^{W}$ 6 | 2 | 0.091 | 0.0586 | 1.47 | 0.129 | 0.0829 | 2.07 |
| $\mathrm{Re}c_{W}^{W}$ 6 | 3 | 0.059 | 0.0662 | 0.926 | 0.083 | 0.0936 | 1.31 |
| $\mathrm{Re}c_{W}^{W}$ 6 | 4 | 0 | 0 | 0 | 0 | 0 | 0 |
| $\mathrm{Re}c_{W}$ 4 | 1 | 0 | 0 | 0 | 0 | 0 | 0 |
| $\mathrm{Re}c_{W}$ 5 | 1 | 0.073 | 0.0272 | 0.625 | 0.082 | 0.0384 | 0.717 |
| $\mathrm{Re}c_{W}$ 5 | 2 | 0.057 | 0.0334 | 0.951 | 0.061 | 0.0472 | 0.096 |
| $\mathrm{Re}c_{W}$ 5 | 3 | 0.092 | 0.0639 | 1.9 | 0.096 | 0.0904 | 1.02 |
| $\mathrm{Re}c_{W}$ 5 | 4 | 0 | 0 | 0 | 0 | 0 | 0 |
