# Supplementary material for: Assessing the benefits of horizontal gene transfer by laboratory evolution and genome sequencing
Source: BMC Evol Biol. 2018 Apr 19;18:54. doi: 10.1186/s12862-018-1164-7 (PMC5909237; doi:10.1186/s12862-018-1164-7)
Supplement: Supplementary file 23 — Text S3. Potentially adaptive gene transfers from the K donor to the W recipient clones during evolution on butyric acid (DOCX 15 kb) [file 12862_2018_1164_MOESM23_ESM.docx]

## S3 Text. Potentially adaptive gene transfers from the K donor to the W recipient clones during evolution on butyric acid

In this analysis, we focused on genes that are present in the *E. coli* K12 but not the W genome, and that were horizontally transferred from the K donor to the W recipient clones during adaptation on butyric acid (59 in total). Among them is the *rhm* operon (homologous to the *yfa* operon in *E. coli* B), which is involved in rhamnose import and metabolism (Badia et al. 1989). One of its genes, *rhmD*, is an enolase with promiscuous substrate specificity to acid sugars [2]. We speculate that acid sugars may be generated during growth on butyric acid that may serve as a substrate for RhmD.

Another transferred operon is the *yfb* operon, which is part of a prophage that also includes the *yfb*, *yfd, yff,* and *yfj* operons [3], which cluster at 2.5 Mb of the *E. coli* K12 genome.

The *yfb* operon has been implicated in the tolerance of *E. coli* to environmental stressors, such as acidity and carbon source limitation [4,5], both of which are relevant for our experiment.

1. Badía J, Baldomà L, Aguilar J, Boronat A. Identification of the rhaA, rhaB and rhaD gene products from Escherichia coli K-12. FEMS Microbiol Lett. The Oxford University Press; 1989;53:253–7.

2. Rakus JF, Fedorov AA, Fedorov E V, Glasner ME, Hubbard BK, Delli JD, et al. Evolution of Enzymatic Activities in the Enolase Superfamily: l-Rhamnonate Dehydratase. Biochemistry. 2008;47:9944–54.

3. Riley M, Abe T, Arnaud MB, Berlyn MKB, Blattner FR, Chaudhuri RR, et al. Escherichia coli K-12: A cooperatively developed annotation snapshot - 2005. Nucleic Acids Res. 2006;34:1–9.

4. Han X, Dorsey-Oresto A, Malik M, Wang J-Y, Drlica K, Zhao X, et al. Escherichia coli genes that reduce the lethal effects of stress. BMC Microbiol. BioMed Central; 2010;10:35.

5. Wang X, Kim Y, Ma Q, Hong SH, Pokusaeva K, Sturino JM, et al. Cryptic prophages help bacteria cope with adverse environments. Nat Commun. Nature Publishing Group; 2010;1:147.
