# Supplementary material for: Assessing the benefits of horizontal gene transfer by laboratory evolution and genome sequencing
Source: BMC Evol Biol. 2018 Apr 19;18:54. doi: 10.1186/s12862-018-1164-7 (PMC5909237; doi:10.1186/s12862-018-1164-7)
Supplement: Supplementary file 25 — Table S9. Number of clones with a mutant allele in specific genes found in the butyric acid-adapted populations. The coordinates of the start and the end of a gene are listed in coordinates of the E. coli W reference genome. α: Number of the of a gene variant sites identified within the given gene among all butyric acid-evolved clones. Β: Number of clones in a given recombination condition (‘$\operatorname{Re}{\mathrm{c}}_{\mathrm{Y}}^{\mathrm{X}}$RecYX’ X: the donor identity; Y: the recipient identity) that harbor derived allele(s) of the given gene. (DOCX 15 kb) [file 12862_2018_1164_MOESM25_ESM.docx]

| Start | End | Gene | Protein | ^α^ Number of variant sites | ^β^ $\mathrm{Re}c_{W}^{B}$ | ^β^ $\mathrm{Re}c_{W}^{K}$ | ^β^ $\mathrm{Re}c_{W}^{W}$ | ^β^ $\mathrm{Re}c_{W}$ |
| --- | --- | --- | --- | --- | --- | --- | --- | --- |
| 715915 | 717378 | citT | citrate:succinate antiporter | 2 | 0 | 0 | 1 | 1 |
| 718837 | 720369 | citF | citrate lyase, citrate-ACP transferase (alpha) subunit | 1 | 0 | 3 | 2 | 1 |
| 829275 | 832076 | sucA | 2-oxoglutarate decarboxylase | 4 | 0 | 0 | 3 | 2 |
| 1031210 | 1033486 | clpA | ATPase and specificity subunit of ClpA-ClpP ATP-dependent serine protease, chaperone activity | 1 | 0 | 0 | 2 | 0 |
| 1259565 | 1259801 | acpP | acyl carrier protein (ACP) | 1 | 0 | 0 | 2 | 0 |
| 2164707 | 2166365 | fliF | flagellar basal-body MS-ring and collar protein | 2 | 2 | 0 | 2 | 0 |
| 2171565 | 2172569 | fliM | flagellar motor switching and energizing component | 1 | 2 | 4 | 3 | 2 |
| 2308733 | 2311180 | gpT2 | tail fiber protein T (tape measure) (GpT) | 4 | 0 | 0 | 4 | 1 |
| 2565117 | 2566319 | ackA | acetate kinase A and propionate kinase 2 | 2 | 0 | 0 | 1 | 2 |
| 2905963 | 2906943 | rluD | 23S rRNA pseudouridine synthase | 1 | 0 | 0 | 2 | 0 |
| 2958838 | 2960376 | emrB | multidrug efflux system protein | 1 | 2 | 0 | 0 | 0 |
| 3191417 | 3192184 | epaR | type III secretion system protein | 1 | 2 | 0 | 0 | 0 |
| 3390075 | 3390902 | cpdA | cyclic 3',5'-adenosine monophosphate phosphodiesterase | 2 | 2 | 0 | 0 | 0 |
| 3529165 | 3531300 | pnp | polynucleotide phosphorylase/polyadenylase | 1 | 0 | 0 | 3 | 2 |
| 3683983 | 3684615 | crp | DNA-binding transcriptional dual regulator | 1 | 2 | 0 | 3 | 2 |
| 4210749 | 4211948 | hemX | uroporphyrinogen III methylase | 1 | 0 | 0 | 3 | 2 |
| 4373890 | 4375398 | glpK | glycerol kinase | 1 | 0 | 0 | 2 | 0 |
| 4445891 | 4449919 | rpoB | RNA polymerase subunit beta | 1 | 0 | 0 | 3 | 2 |
| 4449996 | 4454219 | rpoC | RNA polymerase, beta prime subunit | 3 | 0 | 0 | 2 | 2 |
| 4512502 | 4513617 | malK | fused maltose transport subunit, ATP-binding component of ABC superfamily/regulatory protein | 1 | 0 | 0 | 2 | 0 |
| 4539880 | 4540416 | ssb | single-stranded DNA-binding protein | 9 | 0 | 0 | 0 | 1 |
| 4694190 | 4696133 | cpdB | 2':3'-cyclic-nucleotide 2'-phosphodiesterase | 1 | 0 | 0 | 2 | 0 |
