# Supplementary material for: Assessing the benefits of horizontal gene transfer by laboratory evolution and genome sequencing
Source: BMC Evol Biol. 2018 Apr 19;18:54. doi: 10.1186/s12862-018-1164-7 (PMC5909237; doi:10.1186/s12862-018-1164-7)
Supplement: Supplementary file 27 — Table S3. Summary of strains used in the conjugation assay, their selective markers, and the locations of these markers in the genome. (DOCX 12 kb) [file 12862_2018_1164_MOESM27_ESM.docx]

| Strain | Antibiotics resistance | Gene conferring the resistance phenotype | Genomic location (bp) | Genomic location (minute) |
| --- | --- | --- | --- | --- |
| K donor | *gentamycin* | *trpB* | 1317222 | 28.37 |
|  | *chloramphenicol* | *hyfC* | 2603847 | 56.08 |
| B donor | *gentamycin* | *trpB* | 1314816 | 28.39 |
|  | *chloramphenicol* | *mbhA* | 250000 | 5.51 |
| W donor | *gentamycin* | *trpB* | 1407451 | 28.71 |
|  | *chloramphenicol* | *hyfC* | 2775527 | 56.06 |
| K recipient | *rifampicin* | *rpoB* | 4181245 | 90.8 |
|  | *nalidixic acid* | *gyrB* | 3877705 | 83.53 |
| B recipient | *rifampicin* | *rpoB* | 4160856 | 89.87 |
|  | *nalidixic acid* | *gyrB* | 3838066 | 82.9 |
| W recipient | *rifampicin* | *rpoB* | 4445891 | 83.66 |
|  | *nalidixic acid* | *gyrB* | 4100003 | 83.66 |
