# Supplementary material for: Assessing the benefits of horizontal gene transfer by laboratory evolution and genome sequencing
Source: BMC Evol Biol. 2018 Apr 19;18:54. doi: 10.1186/s12862-018-1164-7 (PMC5909237; doi:10.1186/s12862-018-1164-7)
Supplement: Supplementary file 29 — Table S5. Nutrient concentrations during the evolution experiments. (DOCX 11 kb) [file 12862_2018_1164_MOESM29_ESM.docx]

| 4-Hydroxyphenylacetate (HPA)-adaptation experiment | | |
| --- | --- | --- |
| Days | Original nutrient - glycerol (percent w/v) | Novel nutrient - 4-Hydroxyphenylacetate (percent w/v) |
| 0 - 9 | 0.03 | 0.17 |
| 10 - 19 | 0.02 | 0.18 |
| 21 - 29 | 0.01 | 0.19 |
| 30 - 39 | 0.005 | 0.195 |
| 40 - 49 | 0.002 | 0.198 |
| 50 - 60 | 0 | 0.2 |
| Butyric acid-adaptation experiment | | |
| Days | Original nutrient - glycerol (percent w/v) | Novel nutrient - butyric acid (percent w/v) |
| 0 - 9 | 0.035 | 0.165 |
| 10 - 19 | 0.025 | 0.175 |
| 21 - 29 | 0.015 | 0.185 |
| 30 - 39 | 0.01 | 0.19 |
| 40 - 69 | 0.005 | 0.195 |
| 70 - 94 | 0.001 | 0.199 |
| 95 - 104 | 0.0005 | 0.1995 |
| 105 - 134 | 0.000375 | 0.199625 |
| 135 - 149 | 0.00025 | 0.19975 |
| 150 - 154 | 0.000175 | 0.199825 |
| 155 - 164 | 0.0000625 | 0.1999375 |
| 165 - 175 | 0 | 0.2 |
