# Supplementary material for: Assessing the benefits of horizontal gene transfer by laboratory evolution and genome sequencing
Source: BMC Evol Biol. 2018 Apr 19;18:54. doi: 10.1186/s12862-018-1164-7 (PMC5909237; doi:10.1186/s12862-018-1164-7)
Supplement: Supplementary file 30 — Table S11. Summary of growth parameters of populations evolved in HPA. The table shows mean and standard deviations of growth rate, carrying capacity and area under the growth curve estimated by Growthcurver v0.2.1 at the end of the HPA-adaptation experiment based on three replicate measurements of growth in HPA-supplemented liquid medium and rounded to three significant digits. (DOCX 14 kb) [file 12862_2018_1164_MOESM30_ESM.docx]

| Population | Mean growth rate | Mean carrying capacity | Mean area under the curve | Standard deviation of growth rate | Standard deviation of carrying capacity | Standard deviation of area under the growth curve |
| --- | --- | --- | --- | --- | --- | --- |
| $\mathrm{Re}c_{K}^{W}$ 1 | 0.545 | 0.702 | 27.4 | 0.0858 | 0.0786 | 3.13 |
| $\mathrm{Re}c_{K}^{W}$ 2 | 0 | 0.0233 | 1.07 | 0 | 0.00473 | 0.217 |
| $\mathrm{Re}c_{K}^{W}$ 3 | 0.214 | 0.772 | 26.4 | 0.067 | 0.141 | 3.47 |
| $\mathrm{Re}c_{K}^{B}$ 1 | 0.453 | 0.676 | 24.2 | 0.0586 | 0.0329 | 0.253 |
| $\mathrm{Re}c_{K}^{B}$ 2 | 0.302 | 0.657 | 23.4 | 0.0433 | 0.0917 | 2.17 |
| $\mathrm{Re}c_{K}^{B}$ 3 | 0.416 | 0.767 | 28.5 | 0.120 | 0.107 | 2.93 |
| $\mathrm{Re}c_{K}^{K}$ 1 | 0 | 0.0245 | 1.13 | 0 | 0.00456 | 0.203 |
| $\mathrm{Re}c_{K}^{K}$ 2 | 0 | 0.0206 | 0.944 | 0 | 0.0037 | 0.167 |
| $\mathrm{Re}c_{K}^{K}$ 3 | 0.316 | 0.841 | 29.2 | 0.0321 | 0.0181 | 1.17 |
| $\mathrm{Re}c_{K}$ 1 | 0 | 0.0189 | 0.867 | 0 | 0.00111 | 0.0569 |
| $\mathrm{Re}c_{K}$ 2 | 0.00667 | 0.0238 | 1.16 | 0.0116 | 0.00935 | 0.337 |
| $\mathrm{Re}c_{K}$ 3 | 0 | 0.0193 | 0.89 | 0 | 0.00119 | 0.0606 |
| $\mathrm{Re}c_{K}^{W}$ 4 | 0.491 | 0.541 | 20.9 | 0.0995 | 0.0444 | 1.51 |
| $\mathrm{Re}c_{K}^{W}$ 5 | 0.351 | 0.723 | 25.8 | 0.261 | 0.175 | 4.04 |
| $\mathrm{Re}c_{K}^{W}$ 6 | 0.473 | 0.425 | 15.9 | 0.0699 | 0.0364 | 1.17 |
| $\mathrm{Re}c_{K}^{B}$ 4 | 0.642 | 0.672 | 25.4 | 0.141 | 0.0433 | 1.41 |
| $\mathrm{Re}c_{K}^{B}$ 5 | 0.330 | 0.838 | 30.9 | 0.0377 | 0.00341 | 0.77 |
| $\mathrm{Re}c_{K}^{B}$ 6 | 0.334 | 0.847 | 29.9 | 0.0388 | 0.0176 | 0.65 |
| $\mathrm{Re}c_{K}^{K}$ 5 | 0 | 0.0293 | 1.33 | 0 | 0.0118 | 0.537 |
| $\mathrm{Re}c_{K}$ 5 | 0 | 0.0208 | 0.888 | 0 | 0.00253 | 0.113 |
| $\mathrm{Re}c_{K}$ 6 | 0 | 0.0179 | 0.813 | 0 | 0.00120 | 0.0516 |
