# Supplementary material for: Assessing the benefits of horizontal gene transfer by laboratory evolution and genome sequencing
Source: BMC Evol Biol. 2018 Apr 19;18:54. doi: 10.1186/s12862-018-1164-7 (PMC5909237; doi:10.1186/s12862-018-1164-7)
Supplement: Supplementary file 31 — Table S15. Summary of alignment strategies. The table lists the reference genome(s) we used in whole-genome sequence alignment using Bowtie2 in each of several analyses (columns) described in the methods, and for each adaptation experiment (rows). (DOCX 15 kb) [file 12862_2018_1164_MOESM31_ESM.docx]

| Populations | Recipient strain | Donor strain | Reference genome(s ) used in alignment-based approach to identify HGT genes | Reference genome(s ) used in SNP-based approach to identify HGT genes | Reference genome(s ) used in SNP analyses to identify mutations in genes of recipient background | Reference genome(s ) used in SNP analyses to identify mutations in HGT genes from donor |
| --- | --- | --- | --- | --- | --- | --- |
| $\mathrm{Re}c_{K}^{W}$ | K12 | W | NC_000913.3 and NC_017635.1 jointly | NC_000913.3 NC_017635.1  separately | NC_000913.3 | NC_017635.1 |
| $\mathrm{Re}c_{K}^{B}$ | K12 | B | NC_000913.3 and NC_012967.1 jointly | NC_000913.3 NC_012967.1  separately | NC_000913.3 | NC_012967.1 |
| $\mathrm{Re}c_{K}^{K}$ | K12 | K | NC_000913.3 | NC_000913.3 | NC_000913.3 | NC_000913.3 |
| $\mathrm{Re}c_{K}$ | K12 | - | NC_000913.3 | NC_000913.3 | NC_000913.3 | - |
| $\mathrm{Re}c_{W}^{B}$ | W | B | NC_017635.1 and NC_012967.1 jointly | NC_017635.1 NC_012967.1 separately | NC_017635.1 | NC_012967.1 |
| $\mathrm{Re}c_{W}^{K}$ | W | K | NC_017635.1 and NC_000913.3 jointly | NC_017635.1 NC_000913.3 separately | NC_017635.1 | NC_000913.3 |
| $\mathrm{Re}c_{W}^{W}$ | W | W | NC_017635.1 | NC_017635.1 | NC_017635.1 | NC_017635.1 |
| $\mathrm{Re}c_{W}$ | W | - | NC_017635.1 | NC_017635.1 | NC_017635.1 | - |
