# Supplementary material for: Assessing the benefits of horizontal gene transfer by laboratory evolution and genome sequencing
Source: BMC Evol Biol. 2018 Apr 19;18:54. doi: 10.1186/s12862-018-1164-7 (PMC5909237; doi:10.1186/s12862-018-1164-7)
Supplement: Supplementary file 33 — Table S6. Number of genes suitable to infer horizontally transferred genes for every combination of donor (columns) and recipient (rows) in both the HPA and butyric acid adaptation experiments. We note that ancestral recipient and donor strains of the same backgrounds may differ in DNA sequence at a small number of nucleotide sites as a result of their strain construction. Thus the number of genes suitable for horizontal gene transfer inference are specific for a donor-recipient combination. ND: not determined. (DOCX 12 kb) [file 12862_2018_1164_MOESM33_ESM.docx]

| **Horizontal transfer inference based on sequence coverage information** | | | | |
| --- | --- | --- | --- | --- |
| **Adaptation experiment** |  | K donor | W donor | B donor |
| HPA | K recipient | ND | 3906 | 3893 |
| Butyric acid | W recipient | 3906 | ND | 3821 |
| **Horizontal transfer inference based on single nucleotide polymorphisms** | | | | |
| **Adaptation experiment** |  | K donor | W donor | B donor |
| HPA | K recipient | 1 | 1283 | 997 |
| Butyric acid | W recipient | 1291 | 37 | 2380 |
