# Supplementary material for: Assessing the benefits of horizontal gene transfer by laboratory evolution and genome sequencing
Source: BMC Evol Biol. 2018 Apr 19;18:54. doi: 10.1186/s12862-018-1164-7 (PMC5909237; doi:10.1186/s12862-018-1164-7)
Supplement: Supplementary file 34 — Table S10. Summary of growth parameters of ancestral strains. Growth parameters of the ancestral E. coli K12, B and W donor and recipient measured in liquid media supplemented with HPA, butyric acid, glucose and glucose with tryptophan. Specifically, the table shows mean and standard deviations of growth rate, carrying capacity and area under the growth curve of each ancestor from three replicate measurements and rounded to three significant digits. (DOCX 14 kb) [file 12862_2018_1164_MOESM34_ESM.docx]

| strain | Donor/recipient | nutrient | Mean growth rate | Mean carrying capacity | Mean area under the growth curve | Standard deviation of growth rate | Standard deviation of carrying capacity | Standard deviation of area under the growth curve |
| --- | --- | --- | --- | --- | --- | --- | --- | --- |
| B | donor | Butyric acid | 0 | 0.0036 | 0.159 | 0 | 0.0034 | 0.151 |
| B | donor | HPA | 0 | 0.0009 | 0.0407 | 0 | 0.00106 | 0.0475 |
| B | donor | Glucose | 0 | 0.00500 | 0.222 | 0 | 0.00577 | 0.257 |
| B | donor | Glucose + tryptophan | 0.769 | 338000 | 3.65 | 0.421 | 676000 | 1.68 |
| B | recipient | Glucose | 1.13 | 0.367 | 10.8 | 0.161 | 0.0181 | 0.733 |
| B | recipient | Glucose + tryptophan | 1.1 | 0.218 | 6.27 | 0.0484 | 0.0128 | 0.438 |
| K | donor | Butyric acid | 0.0002 | 0 | 0.08 | 0.000400 | 0 | 0.0561 |
| K | donor | HPA | 0 | 0.0016 | 0.0733 | 0 | 0.00206 | 0.0918 |
| K | donor | Glucose | 0 | 0.0055 | 0.241 | 0 | 0.00332 | 0.152 |
| K | donor | Glucose + tryptophan | 0.0982 | 676000 | 0.548 | 0.0659 | 469000 | 0.375 |
| K | recipient | HPA | 0 | 0.0061 | 0.27 | 0 | 0.00415 | 0.184 |
| K | recipient | Glucose | 1.15 | 0.531 | 14.5 | 0.17 | 0.0495 | 0.929 |
| K | recipient | Glucose + tryptophan | 0.904 | 0.394 | 10.2 | 0.0807 | 0.0521 | 2.01 |
| W | donor | Butyric acid | 0 | 0.00100 | 0.0446 | 0 | 0.000660 | 0.0305 |
| W | donor | HPA | 0 | 0.00170 | 0.0741 | 0 | 0.000520 | 0.0242 |
| W | donor | Glucose | 0 | 0.00280 | 0.124 | 0 | 0.00443 | 0.199 |
| W | donor | Glucose + tryptophan | 0.254 | 370000 | 1.75 | 0.279 | 428000 | 2.18 |
| W | recipient | Butyric acid | 0 | 0.00380 | 0.168 | 0 | 0.00027 | 0.0133 |
| W | recipient | Glucose | 1.18 | 0.438 | 11.4 | 0.0838 | 0.0296 | 1.69 |
| W | recipient | Glucose + tryptophan | 0.577 | 829000 | 2.81 | 0.5 | 957000 | 1.79 |
