# Supplementary material for: Assessing the benefits of horizontal gene transfer by laboratory evolution and genome sequencing
Source: BMC Evol Biol. 2018 Apr 19;18:54. doi: 10.1186/s12862-018-1164-7 (PMC5909237; doi:10.1186/s12862-018-1164-7)
Supplement: Supplementary file 35 — Table S4. Nutrient concentrations tested for cross-feeding experiments. (DOCX 11 kb) [file 12862_2018_1164_MOESM35_ESM.docx]

| Recipient | Donor | 4-Hydroxyphenylacetate (HPA) / Butyric acid concentration (percent w/v) | Glycerol concentration (percent w/v) |
| --- | --- | --- | --- |
| K | B/K/W | 0.15 | 0.05 |
| K | B/K/W | 0.17 | 0.03 |
| K | B/K/W | 0.185 | 0.0015 |
| K | B/K/W | 0.2 | 0 |
| W | B/K/W | 0.15 | 0.05 |
| W | B/K/W | 0.17 | 0.03 |
| W | B/K/W | 0.185 | 0.0015 |
| W | B/K/W | 0.2 | 0 |
